# Solanimycin: Biosynthesis and Distribution of a New Antifungal Antibiotic Regulated by Two Quorum-Sensing Systems

**DOI:** 10.1128/mbio.02472-22

**Published:** 2022-10-10

**Authors:** Miguel A. Matilla, Rita E. Monson, Annabel Murphy, Muriel Schicketanz, Alison Rawlinson, Caia Duncan, Juan Mata, Finian Leeper, George P. C. Salmond

**Affiliations:** a Department of Biochemistry, University of Cambridge, Cambridge, United Kingdom; b Department of Environmental Protection, Estación Experimental del Zaidín, Consejo Superior de Investigaciones Científicas, Granada, Spain; c Yusuf Hamied Department of Chemistry, University of Cambridge, Cambridge, United Kingdom; University of Wisconsin-Madison

**Keywords:** *Dickeya solani*, acyl-homoserine lactone, agriculture and global food security, antibiotics, antifungal agents, gene regulation, horizontal gene transfer, hybrid polyketide/nonribosomal peptide, phytopathogens, post-transcriptional control mechanisms, quorum sensing, secondary metabolism

## Abstract

The increasing emergence of drug-resistant fungal infections has necessitated a search for new compounds capable of combating fungal pathogens of plants, animals, and humans. Microorganisms represent the main source of antibiotics with applicability in agriculture and in the clinic, but many aspects of their metabolic potential remain to be explored. This report describes the discovery and characterization of a new antifungal compound, solanimycin, produced by a hybrid polyketide/nonribosomal peptide (PKS/NRPS) system in Dickeya solani, the enterobacterial pathogen of potato. Solanimycin was active against a broad range of plant-pathogenic fungi of global economic concern and the human pathogen Candida albicans. The genomic cluster responsible for solanimycin production was defined and analyzed to identify the corresponding biosynthetic proteins, which include four multimodular PKS/NRPS proteins and several tailoring enzymes. Antifungal production in D. solani was enhanced in response to experimental conditions found in infected potato tubers and high-density fungal cultures. Solanimycin biosynthesis was cell density dependent in D. solani and was controlled by both the ExpIR acyl-homoserine lactone and Vfm quorum-sensing systems of the bacterial phytopathogen. The expression of the solanimycin cluster was also regulated at the post-transcriptional level, with the regulator RsmA playing a major role. The solanimycin biosynthetic cluster was conserved across phylogenetically distant bacterial genera, and multiple pieces of evidence support that the corresponding gene clusters were acquired by horizontal gene transfer. Given its potent broad-range antifungal properties, this study suggests that solanimycin and related molecules may have potential utility for agricultural and clinical exploitation.

## INTRODUCTION

Many important drugs for treating microbial infections are derived from natural products produced by microorganisms (1, 2). In an era of increasing antimicrobial resistance, there is an urgent need for discovery of new antibiotics for use in medicine and agriculture (3–5). Several antifungal drugs derived from natural products, or that mimic natural products, have been approved (2, 6). However, the number of natural product-derived antifungal antibiotics developed in the last 40 years is significantly lower than that of their antibacterial counterparts (2).

Pathogenic fungi represent a major worldwide threat to agriculture and global food security. Data from the Food and Agriculture Organization of the United Nations (FAO) indicate that plant pathogens cause losses of up to 40% in annual crop production, with fungal phytopathogens responsible for most of these crop losses (7–9). Furthermore, these phytopathogens can be responsible for up to 80% of total loss under disease-conducive conditions (10). This global health problem has been exacerbated by monoculture cropping practices, the loss of crop diversity due to intensive agriculture, ecological deterioration of seminatural landscapes, and the anthropogenic spread of fungal phytopathogens into favorable habitats and naive hosts (3, 10). Close to 80% of fungicides currently used in agriculture are single-target antimicrobials, with azoles, strobilurins, and succinate dehydrogenase inhibitors accounting for ~60% of the global market (10). The rapid emergence of resistance to these major classes of fungicides necessitates identification of novel broad-spectrum antifungals with new mechanisms of action (3, 10).

Most bioactive natural products from bacteria are encoded in biosynthetic gene clusters (11, 12), with some bacteria dedicating up to ~14% of their genomes to the synthesis of these secondary metabolites (13, 14). Polyketides (PKs) and nonribosomal peptides (NRPs) are two of the largest families of secondary metabolites with a broad range of biological activities, including antibacterial, antifungal, immunosuppressant, and anticancer activity, among others (15). PKs and NRPs are synthesized by polyketide synthases (PKSs) and nonribosomal peptide synthetases (NRPSs), respectively. These PKS and NRPS machineries are typically organized in multidomain modules that, through the condensation of carboxylic or amino acids, assemble many structurally and functionally diverse PKs and NRPs, respectively (15). The final PK and NRP products are often modified by specialized tailoring enzymes, a process that largely contributes to the diversification and biological activities of the final metabolite (16, 17).

Historically, soil actinomycetes have been the main source of bioactive secondary metabolites currently used in the clinic and agriculture (5, 18, 19). However, developments in genomics, metagenomics, proteomics, genome mining, and analytical chemistry approaches are revealing that alternative bacterial taxa, including plant-associated bacteria, are rich sources of secondary metabolites that might be exploited in chemotherapeutic drug discovery and agriculture (14, 20–22). Indeed, a recent analysis of ~217,000 bacterial genomes and metagenome-assembled genomes revealed that only ~3% of the bacterial genetic potential for the biosynthesis of secondary metabolites has been explored experimentally, and plant-associated proteobacteria were identified as strong candidates for the identification of novel bioactive natural products (23).

Our earlier work focused on the emerging phytopathogen Dickeya solani, previously classified as Pectobacterium chrysanthemi (Erwinia chrysanthemi) (24, 25). D. solani was first reported on tomato in 2005 to 2006, but subsequent reports of this organism revealed that it had been widespread around Europe and Israel (24, 26). It primarily causes blackleg disease in potato plants but is thought to have crossed over from ornamental plants, as its first isolation was on hyacinth (24, 25). D. solani has now been prioritized as one of the top 10 bacterial plant pathogens of concern (27). However, D. solani is also of interest because it carries several different secondary metabolite gene clusters; some of which are primarily associated with *Dickeya* spp. and not found in other taxonomically related plant pathogens (28–30). Among them, the hybrid PK/NRP zeamine that is toxic to fungi, bacteria, and nematodes (31–33), the antifungal and antioomycete polyketide oocydin A (34, 35), and the purple pigment indigoidine (36) are all secondary metabolites produced by species in the genus *Dickeya*, indicating that *Dickeya* spp. could be important reservoirs of novel, and potentially useful, natural products.

While examining natural product biosynthesis in D. solani, we noticed that several mutant strains defective in the production of oocydin A lost their antioomycete properties but still retained strong activity against several plant-pathogenic fungi such as Verticillium dahliae, suggesting that these strains produced another, uncharacterized, antifungal antibiotic. Here, multidisciplinary approaches were used to define and characterize a biosynthetic gene cluster responsible for the residual but potent broad-spectrum antifungal activity. The regulation of its biosynthetic locus was interrogated and found to be modulated by multiple environmental cues and regulatory pathways.

## RESULTS

### Dickeya solani produces a previously undiscovered second antifungal compound.

**FIG 1:**
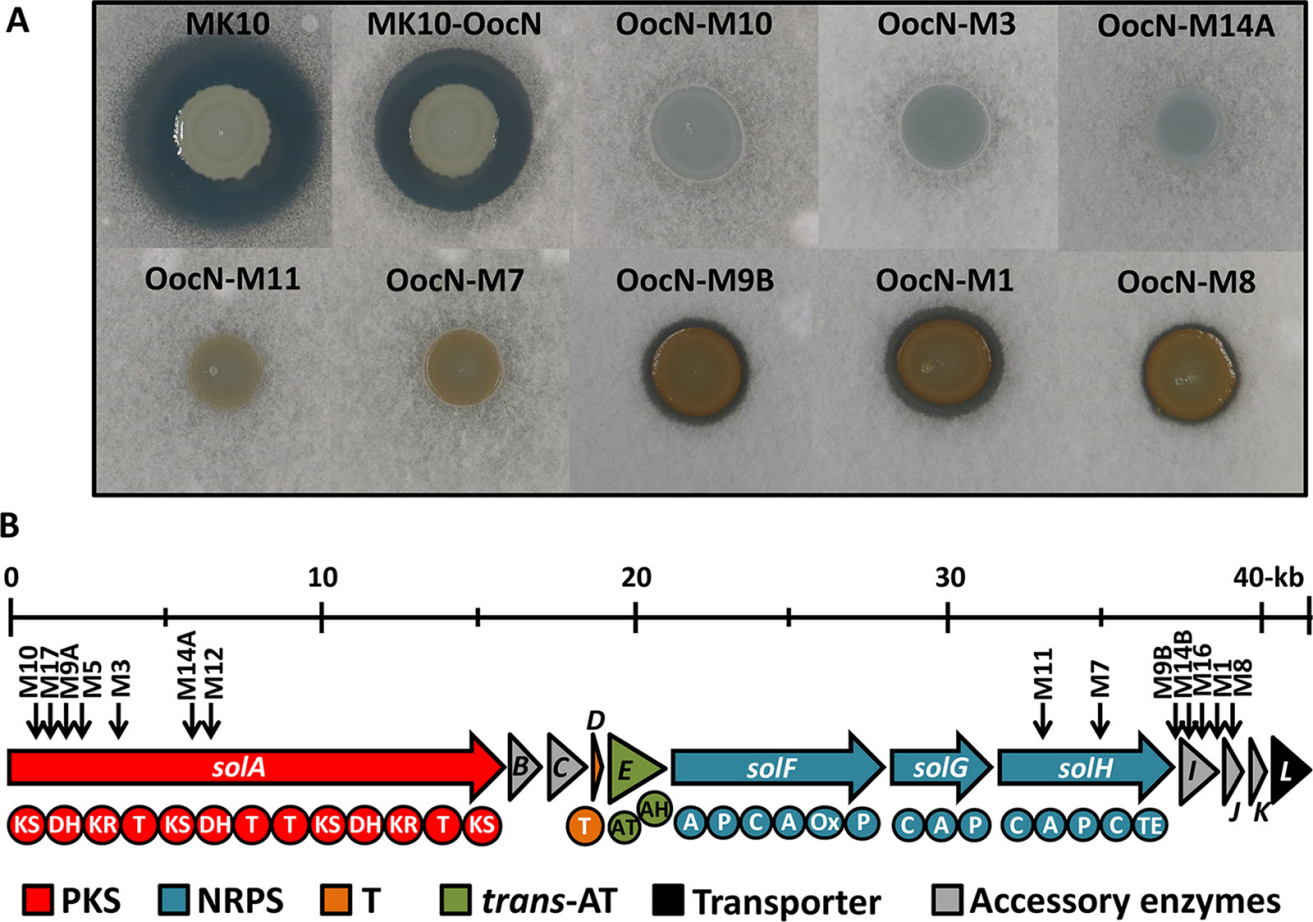
Identification and characterization of a novel hybrid PKS/NRPS antifungal gene cluster in Dickeya solani MK10. (A) Antifungal activity against Verticillium dahliae of MK10 and derivative strains with mutations in the oocydin A and the solanimycin (*sol*) biosynthetic clusters. The size of the inhibition halos is indicative of the susceptibility of Verticillium dahliae, present in the top agar lawns, to the antifungal antibiotics produced by Dickeya solani MK10, oocydin A, and solanimycin. The bioassays were repeated at least three times, and representative results are shown. Pictures were taken after 96 h of incubation at 25°C. Genotypic characteristics of the bacterial strains used are detailed in Table S1 in the supplemental material. (B) Genetic organization of the *sol* gene cluster in MK10. Locations of the transposon insertions are shown by black arrows with the indicated strain names above. Color code representing the functional category of each gene of the gene cluster is given. T, acyl carrier protein; KS, ketosynthase; DH, dehydratase; KR, ketoreductase; C, condensation; A, adenylation; P, peptidyl carrier protein; Ox, oxidase; TE, thioesterase; AT, acyltransferase; and AH, acyl hydrolase.

During the characterization of the bioactive properties of transposon insertion mutants in D. solani MK10, we observed that although oocydin A-deficient mutants (i.e., MK10-OocF, MK10-OocG, and MK10-OocN) had lost their ability to antagonize the growth of a phytopathogenic oomycete (see Fig. S1A in the supplemental material), they remained antagonistic to plant-pathogenic fungi such as Verticillium dahliae (Fig. 1A). To determine whether this phenotype was exclusive to strain MK10, we used the phage ϕXF1 to transduce random transposon mutations in the oocydin A gene cluster into other oocydin A-producing D. solani strains (34). As observed for MK10, oocydin A-deficient mutants of D. solani strains MK16 and IPO_2222 still exhibited strong antifungal activities (Fig. S1B), reinforcing the notion that another antifungal compound was being produced by these three D. solani strains.

10.1128/mbio.02472-22.2 FIG S1 Antioomycete, antifungal, and nematicide activities of the Dickeya solani strains with mutations in the oocydin A and solanimycin biosynthetic gene clusters. (A) Bioactivity of Dickeya solani MK10 and derivative mutant strains defective in the oocydin A gene cluster against the oomycete Pythium ultimum. (B) Antifungal activity against Verticillium dahliae of Dickeya solani strains MK16 and IPO_2222 and derivative strains with mutations in the oocydin A biosynthetic gene cluster. (C) Antifungal activity against Verticillium dahliae of Dickeya solani MK16 and derivative strains defective in the synthesis of oocydin A (MK16-OocN) and double mutants in the oocydin A and solanimycin gene clusters (16OocN-M14, 16OocN-M10, 16OocN-M16, and 16OocN-M11). (D) Antifungal activity against S. pombe of different *Dickeya* strains. The number above each spot indicates the corresponding strain, listed on the side. In panels A to D, the bioassays were repeated at least three times, and representative results are shown. (E) Survival of Caenorhabditis elegans when cultured on different strains of Dickeya solani MK10. The results of a representative trial with at least 50 worms under each condition are shown. The nonpathogenic strain Escherichia coli OP50 was used as negative control. In panels A to D, the size of the inhibition halos is indicative of the susceptibility of the tested fungi and oomycete to solanimycin. Genotypic characteristics of the strains used are detailed in Table S1. Download FIG S1, JPG file, 0.8 MB. Copyright © 2022 Matilla et al. 2022 Matilla et al. https://creativecommons.org/licenses/by/4.0/ This content is distributed under the terms of the Creative Commons Attribution 4.0 International license.

10.1128/mbio.02472-22.1 TABLE S1 Bacteria, oomycetes, fungi, phages, plasmids, and oligonucleotides used in this study. Download Table S1, DOCX file, 0.1 MB. Copyright © 2022 Matilla et al. 2022 Matilla et al. https://creativecommons.org/licenses/by/4.0/ This content is distributed under the terms of the Creative Commons Attribution 4.0 International license.

### A novel hybrid NRPS/PKS gene cluster is responsible for the synthesis of the uncharacterized antifungal antibiotic.

To identify the genes involved in the strong residual antifungal activities, the oocydin A-deficient mutant MK10-OocN was used to screen for random transposon mutants totally defective in bioactivity against V. dahliae. This screening allowed the isolation of multiple independent transconjugants showing complete loss or reduced antifungal properties (Fig. 1A). To confirm the association between the transposon insertion and the loss of the antifungal activity, all the mutations were transduced back into the strain MK10-OocN. Additionally, several mutations were also transduced into the oocydin A-negative mutant of D. solani MK16, MK16-OocN. As expected, the resulting transductants showed complete loss of the antifungal properties of MK16 toward V. dahliae (Fig. S1C).

The locations of the transposons in MK10-OocN were determined and all insertions mapped to a 40.3-kb uncharacterized hybrid NRPS/PKS gene cluster (Fig. 1B). This genetic locus was thus required for production of the uncharacterized antifungal, which we designated solanimycin. In D. solani MK10, the solanimycin (*sol*) biosynthetic cluster is composed of 12 open reading frames (ORFs), and *in silico* analyses allowed us to assign putative biosynthetic roles to each ORF (Table 1). The *sol* cluster encodes a large multimodular type I PKS (SolA), 3 multidomain NRPSs (SolF, SolG, and SolH), and an orphan acyl carrier protein (ACP) (SolD) (Fig. 1B; Table 1). No acyltransferase (AT) domains were identified in the PKS biosynthetic modules of SolA, and, in accordance with the *trans*-AT nature of SolA, the biosynthetic cluster encodes a freestanding AT (SolE) containing two putative AT domains (Fig. 1B; Table 1). Several putative tailoring enzymes, potentially involved in the chemical modification of the nonribosomal peptide backbone during or after chain elongation, were also identified within the biosynthetic cluster, including a NAD-dependent epimerase/dehydratase (SolB), an aminotransferase (SolC), a cytochrome P450 (SolI), a putative metallo-hydrolase (SolJ), and a hydratase (SolK). The last gene of the biosynthetic cluster is predicted to encode a multidrug antimicrobial extrusion protein (SolL), potentially involved in the secretion of the metabolite to the extracellular environment (Fig. 1B; Table 1).

**TABLE 1:** Deduced functions of ORFs in Dickeya solani MK10 solanimycin biosynthetic gene cluster

Protein	Size (aa)	Proposed function^*a*^	Sequence similarity (protein, origin)	Identity/similarity (%)	GenPept accession no.
SolA	5,358	PKS (KS-DH-KR-T-KS-DH-T-T-KS-DH-KR-T-KS)	LT85_1869, Collimonas arenae	50/64	AIY41027
SolB	329	NAD-dependent epimerase/dehydratase	LT85_1870, Collimonas arenae	58/73	AIY41028.1
SolC	415	Aminotransferase	GLE_2101, Lysobacter enzymogenes	77/89	ALN57451
SolD	89	Acyl carrier protein (T)	GLE_2102, Lysobacter enzymogenes	55/72	ALN57452
SolE	628	Acyltransferase (AT-AH)	LT85_1874, Collimonas arenae	56/70	AIY41032
SolF	2,300	NRPS (A-P-C-A-ox-P)	WI73_12820, Burkholderia ubonensis	47/50	KVC70826
SolG	1,163	NRPS (C-A-P)	RBRH_00484, Paraburkholderia rhizoxinica	58/72	CBW76566
SolH	1,908	NRPS (C-A-P-C-TE)	WP_132343500, Photorhabdus luminescens	47/63	WP_132343500
SolI	428	Cytochrome P450	ABS77_08630, *Phenylobacterium* sp. strain SCN 69-14	59/76	ODT61829
SolJ	241	Metallo-hydrolase	AWB69_02897, Caballeronia udeis	48/62	SAL32886
SolK	180	Enoyl-CoA hydratase	Pden_4680, Paracoccus denitrificans PD1222	54/76	ABL72741
SolL	447	MATE family efflux transporter	WP_211266708, Halotalea alkalilenta	53/72	WP_211266708

a T, acyl carrier protein; KS, ketosynthase; DH, dehydratase; KR, ketoreductase; C, condensation; A, adenylation; P, peptidyl carrier protein; Ox, oxidase; TE, thioesterase; AT, acyltransferase; AH, acyl hydrolase.

### The solanimycin biosynthetic cluster is widely distributed within *Dickeya* species and other enterobacteria.

**FIG 2:**
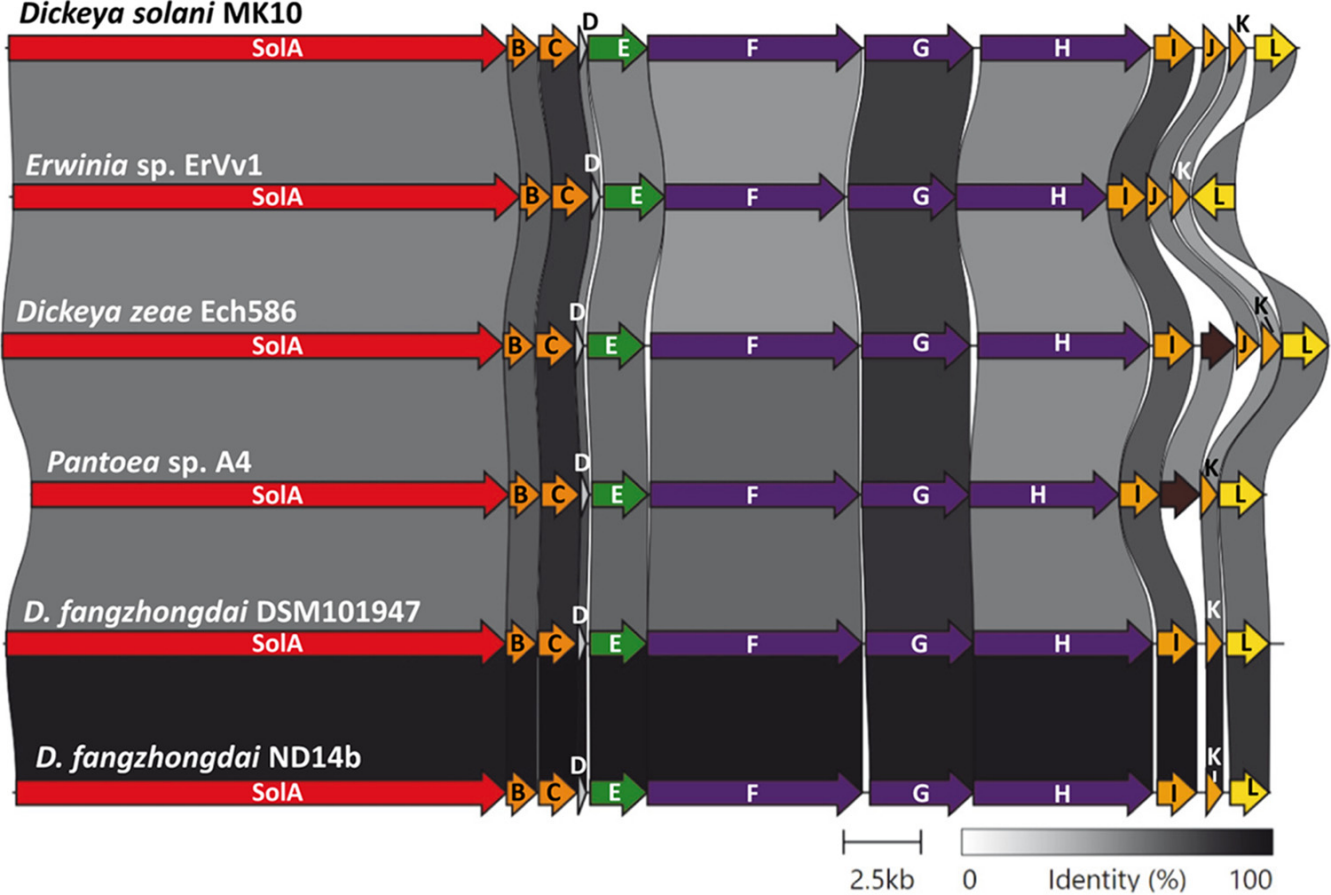
Homology between the solanimycin gene cluster of D. solani MK10 and the biosynthetic clusters of other enterobacterial strains. The alignments show a comparison of homology between the *sol* gene cluster of MK10 and the gene clusters of *Erwinia* sp. ErVv1, D. zeae Ech586, *Pantoea* sp. A4, D. fangzhongdai DSM 101947, and D. fangzhongdai ND14b. The percentages of amino acid translation identities between the different *sol* clusters are indicated in gray according to the scale present. Biosynthetic clusters were ordered to highlight the diversity in the downstream region of the gene cluster. Alignments were generated using clinker (92).

The genomes of 382 *Dickeya* spp. were interrogated to assess the distribution of the *sol* biosynthetic cluster. Just over 100 of the *in silico*-analyzed *Dickeya* strains carry the *sol* cluster. These include D. solani, D. zeae, D. dadantii, D. aquatica, and D. fangzhongdai species and multiple unclassified *Dickeya* strains (Fig. 2 and Fig. S2). We assessed the antifungal activity of 15 *Dickeya* strains available in our laboratory stocks, and we detected antifungal activity attributable to solanimycin in half of the strains (Fig. S1D), mirroring the genomic predictions.

10.1128/mbio.02472-22.3 FIG S2 Homology between the solanimycin gene cluster of D. solani MK10 and the biosynthetic clusters of the other enterobacterial strains. The alignments show a comparison of homology between the *sol* gene cluster of D. solani MK10 and the gene clusters of D. solani MK16, D. solani IPO2222, D. solani IFB0099, D. aquatica 174/2, D. dadantii NCPPB3537, D. zeae NCPPB3531, D. zeae MS1, Rouxiella chamberiensis 130333, R. badensis DSM 100043, and *Gammaproteobacteria* bacterium WG36. The percent amino acid translation identities between the different *sol* clusters are indicated in gray according to the scale at the bottom right. Biosynthetic clusters were reordered to highlight the diversity in the downstream region of the gene cluster and to order D. solani MK10 at the top as a reference. Download FIG S2, JPG file, 1.1 MB. Copyright © 2022 Matilla et al. 2022 Matilla et al. https://creativecommons.org/licenses/by/4.0/ This content is distributed under the terms of the Creative Commons Attribution 4.0 International license.

Further bioinformatic analyses revealed that the *sol* cluster is not restricted to the *Dickeya* genus, as it was also identified in the plant-associated strains, *Pantoea* sp. strain A4 and *Erwinia* species strains ErVv1 and AG740 (NCBI assembly accession nos. GCA_000295955, GCA_900068895, and GCA_003201495, respectively) (Fig. 2). Furthermore, the *sol* biosynthetic cluster is also present in Rouxiella chamberiensis 130333 (GenBank accession no. NZ_JRWU00000000) and Rouxiella badensis DSM 100043 (GenBank accession no. NZ_MRWE00000000) and partially within gamma proteobacterium WG36 (GenBank accession no. AMYV00000000.1) and Teredinibacter turnerae T7901 (GenBank accession no. NC_012997) (Fig. S2), bacterial isolates from a parenteral nutrition bag in a French hospital, a German peat bog, Michigan’s Wintergreen Lake, and a wood-boring mollusk, respectively.

Comparative analyses define that the *sol* biosynthetic clusters are between 39.7 and 42.8 kb, and they are between 65.4% and 100% identical at the DNA level to the gene cluster of MK10 (Fig. 2 and Fig. S2). Several pieces of evidence support that the *sol* clusters were acquired by horizontal gene transfer (HGT) between different genera and species of bacteria, including: (i) remnant sequences of integrases and transposases as well as phage genes (e.g., holins, lysozymes, and lysis regulatory proteins) were identified bordering the clusters in multiple *Dickeya* strains; and (ii) while the genomic context differed between different strains, in many *Dickeya* spp., the same three tRNAs, *glyW*, *cysT*, and *leuZ*, flanked the *sol* region (Fig. S3). tRNAs genes were defined as hot spots for the integration of genes in HGT events (37), and we noticed that different secondary metabolite clusters were present at the same site in different *Dickeya* strains (Fig. S3).

10.1128/mbio.02472-22.4 FIG S3 Horizontal gene transfer events exist at the *glyW-cysT-leuZ* site in different genomes. The genomic contexts of the *glyW-cysT-leuZ* site in Escherichia coli, D. solani, D. dianthicola, Serratia marcescens, D. dadantii, and D. paradisiaca are indicated in green. The size of any insertion in the *glyW-cysT-leuZ* site is indicated, and any inserted genes are shown, grouped in color by predicted function. Download FIG S3, JPG file, 1.0 MB. Copyright © 2022 Matilla et al. 2022 Matilla et al. https://creativecommons.org/licenses/by/4.0/ This content is distributed under the terms of the Creative Commons Attribution 4.0 International license.

### Different gene configurations between solanimycin gene clusters.

**FIG 3:**
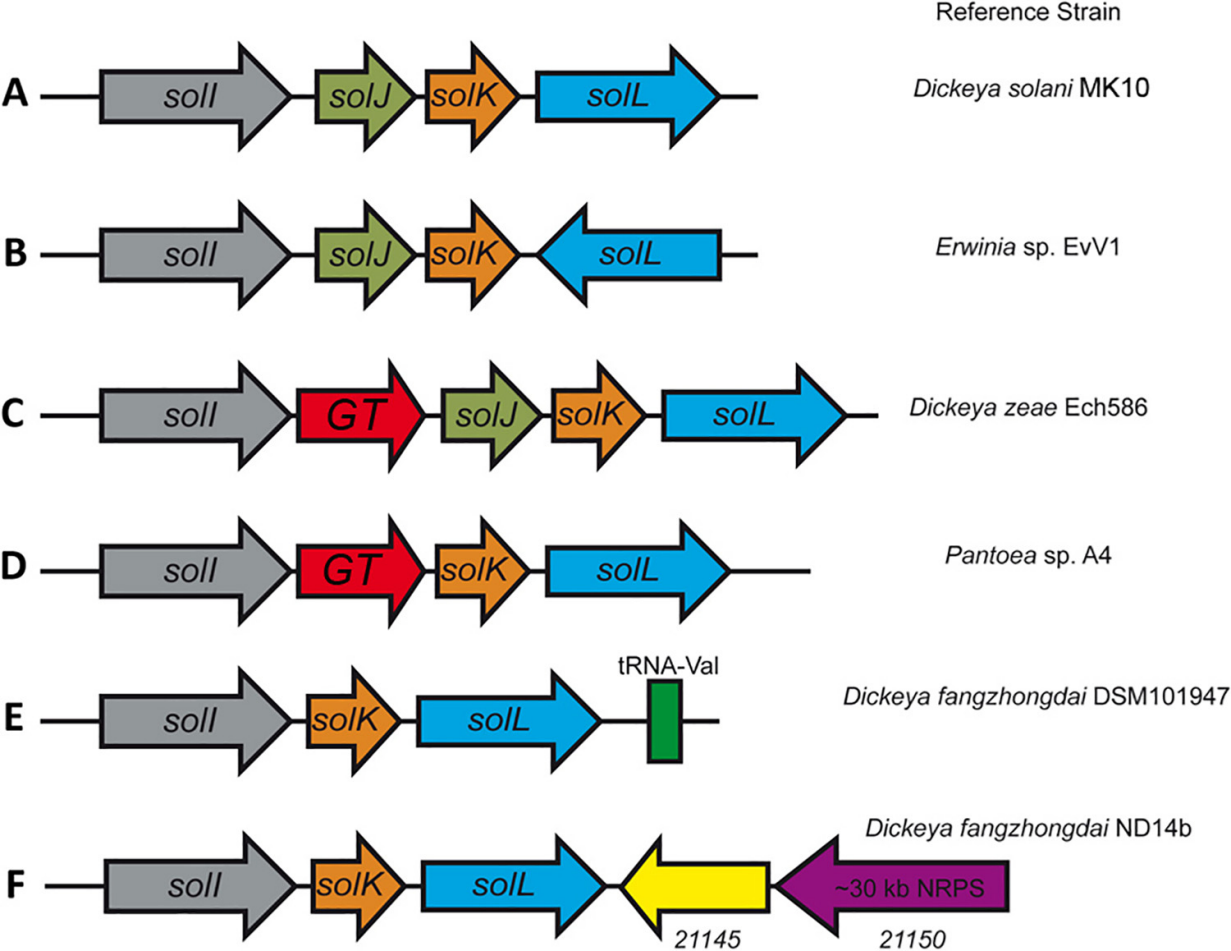
Genetic organization of the downstream region of the solanimycin gene cluster. For each configuration, a reference genome is indicated to the right. Configurations shown are D. solani MK10 (A), *Erwinia* sp. ErVv1 (B), D. zeae Ech586 (C), *Pantoea* sp. A4 (D), D. fangzhongdai DSM 101947 (E), and D. fangzhongdai ND14b (F). Where appropriate, the gene number is indicated below ORFs falling outside the immediate *sol* gene cluster. Color code represents the functional category of each gene, including cytochrome P450 SolI (gray), metallo-hydrolase SolJ (green), enoyl-CoA hydratase SolK (orange), MATE transporter SolL (blue), glycosyltransferase (GT) family I (red), NRPS (purple), and cyclic peptide transporter (yellow).

Six different gene configurations were identified between the downstream regions of the *sol* gene clusters (Fig. 2 and 3). The most common genetic organization was identified in MK10 (Fig. 3A). The same organization was found in *Erwinia* sp. ErVv1, although the gene *solL* was convergently transcribed with the rest of the biosynthetic operon (Fig. 3B). Surprisingly, a gene encoding a glycosyltransferase was downstream of the *solI* gene in several strains, for example, D. zeae Ech586 and R. chamberiensis 130333 (Fig. 3C and Fig. S2). This glycosyltransferase-encoding gene was also present in the biosynthetic cluster from *Pantoea* sp. A4, although the *solJ* gene was absent in this strain (Fig. 2 and Fig. 3D). The *solJ* gene was also absent from the biosynthetic clusters from D. fangzhongdai strains ND14b and DSM 101947 (Fig. 3E and F), as well as *Dickeya* species strains MK7, NCPPB_3274, B16, and M005. Curiously, the *sol* cluster in D. fangzhongdai ND14b was followed by an additional 30-kb ORF predicted to encode an NRPS (Fig. 3F).

Comparative analysis of the NRPSs SolF, SolG, and SolH revealed unexpected results between different strains containing a *sol* biosynthetic cluster. Surprisingly, SolF is missing approximately 500 amino acids, predicted to encode the monooxygenase domain, in *Dickeya* species strains FVG1-MFV-017 and FVG-MFV-A16. In addition, SolH in the biosynthetic clusters from *Pantoea* sp. A4 and *Erwinia* sp. ErVv1 lacked the final thioesterase domain and is around 300 amino acids shorter than SolH proteins from the remaining *sol* clusters (Fig. 2 and Fig. S2). In contrast, SolG was highly conserved in all strains. Thus, while there is broad conservation across the *sol* biosynthetic clusters, significant differences exist between individual producers, implying that some chemical diversity of secondary metabolites may be elaborated across the different strains.

### Contribution of individual *sol* genes to solanimycin production.

**FIG 4:**
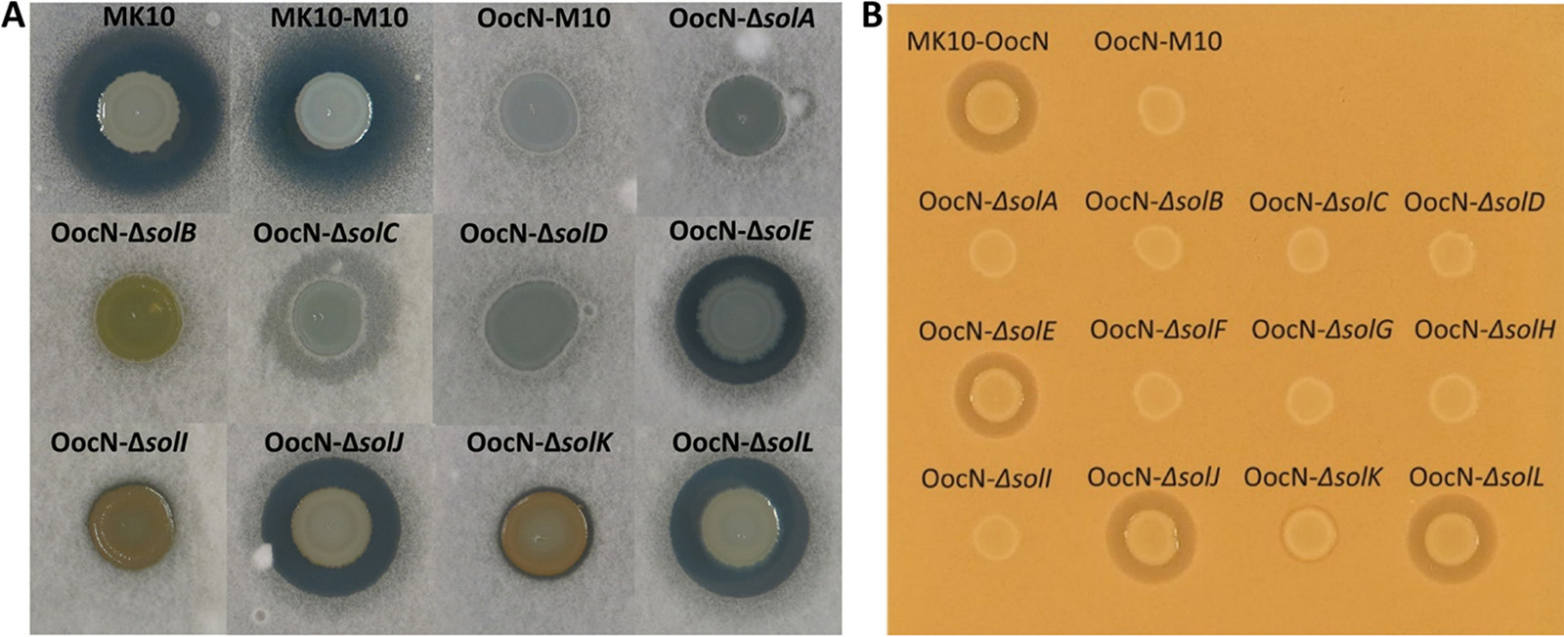
Antifungal properties of individual gene knockouts in the solanimycin biosynthetic cluster. Bioactivities of MK10 and strains deleted for individual genes in the *sol* cluster (*solA* to *solL*) were assayed on a Verticillium dahliae (A) and Schizosaccharomyces pombe (B) top lawns. The size of the inhibition halos is indicative of the susceptibility of the tested fungi, which are present in the top agar lawns, to solanimycin. Genotypic characteristics of the strains used are detailed in Table S1 in the supplemental material. Bioassays were imaged after 72 to 96 h of incubation at 25°C. The bioassays were repeated at least three times, and representative pictures are shown.

Curiously, all the transposon insertions blocking solanimycin production in the isolated mutants were in the upstream and downstream ends of the *sol* cluster, with no solanimycin-negative insertions between *solB* and *solE* (Fig. 1B). To assess whether the *sol* cluster comprised a single transcriptional unit, analysis of MK10 RNA transcripts by reverse transcriptase PCR (RT-PCR) was undertaken. Products were detected across each intergenic region in the biosynthetic cluster but not upstream of the cluster or between the 12th gene and the ORF immediately downstream (Fig. S4). Thus, we concluded that insertions in the first gene in the cluster would have polar effects on downstream genes, consistent with operonic organization. Surprisingly, transposon insertions in the downstream end of the gene cluster resulted in the acquisition of an orange pigmentation when the mutant strains were grown in potato dextrose (PD) medium (Fig. 1A). To gain insights into solanimycin biosynthesis, in-frame deletions in all 12 genes in the cluster were constructed by allelic exchange. The antifungal activities of these mutants were characterized (Fig. 4), and, where possible due to size, mutants were functionally complemented in *trans* (Fig. S5A).

10.1128/mbio.02472-22.5 FIG S4 The *sol* biosynthetic gene cluster consists of a large polycistronic unit. This figure shows transcript analysis by RT-PCR using primers designed to span the intergenic region between two adjacent genes. For each region, three PCR analyses were carried out: +, RT-PCR on cDNA; −, negative control with no reverse transcriptase; and DNA, positive control with genomic DNA as the template. Download FIG S4, JPG file, 0.5 MB. Copyright © 2022 Matilla et al. 2022 Matilla et al. https://creativecommons.org/licenses/by/4.0/ This content is distributed under the terms of the Creative Commons Attribution 4.0 International license.

10.1128/mbio.02472-22.6 FIG S5 Genetic and chemical complementation of Dickeya solani MK10 mutants. (A) Genetic complementation of mutants mapping to the *sol* gene cluster. Bioactivity against S. pombe are shown. Pictures were taken after 48 incubation at 25°C. (B) Genetic and chemical complementation of an *expI* mutant. Bioactivity against S. pombe is shown. In all cases, induction of protein expression was done by addition of 1 mM of IPTG. The size of the inhibition halos is indicative of the susceptibility of the tested fungi, which are present in the top agar lawns, to solanimycin. Genotypic characteristics of the strains used are detailed in Table S1. Download FIG S5, JPG file, 1.3 MB. Copyright © 2022 Matilla et al. 2022 Matilla et al. https://creativecommons.org/licenses/by/4.0/ This content is distributed under the terms of the Creative Commons Attribution 4.0 International license.

The core biosynthetic machinery of the antifungal metabolite consists of a PKS (SolA) and three NRPSs (SolF, SolG, and SolH) (Fig. 1B), and deletion of any one of these genes resulted in loss of antifungal activity (Fig. 4). Deletion of the freestanding ACP-encoding gene *solD* caused complete loss of antifungal activity (Fig. 4), indicating the essential role of this protein for antibiotic biosynthesis. The *trans*-AT SolE contains two AT domains (SolE-AT_1_ and SolE-AT_2_), both containing the catalytic Ser-His dyad and the conserved N-terminal GQGSP loop (Fig. S6). *In silico* analyses revealed that SolE-AT_1_ possesses the conserved residues characteristic of malonyl-specific ATs. However, these residues are less conserved in SolE-AT_2_ (Fig. S6). Multiple-sequence alignments revealed that the characteristics of malonyl-specific ATs are also poorly conserved in PedC and KirCl-AT_1_ (Fig. S6). PedC and KirCl-AT_1_ have no AT activities and are acyl hydrolases suggested to act as PKS proofreading enzymes to release stalled biosynthetic intermediates (38, 39) as an indication that SolE-AT_2_ may play a secondary role in the biosynthetic pathway of solanimycin. Surprisingly, the deletion of *solE* did not diminish the antifungal properties of MK10-OocN (Fig. 4), implying that another enzyme with AT activity may be encoded in the genome of MK10. In accordance with this notion, three freestanding AT enzymes were identified in the MK10 genome, DSOMK10_RS0113975, OocV, and OocW.

10.1128/mbio.02472-22.7 FIG S6 Multiple-sequence alignment of *trans-*acyltransferase (AT) domains. S-H catalytic dyads are shown in red. AT active sites (blue), together with additional conserved residues (yellow) for malonate-specific domains and N-terminal GQGSP loop (underlined) based on Yadav and coworkers (Yadav G, Gokhale RS, Mohanty D. 2003. Computational approach for prediction of domain organization and substrate specificity of modular polyketide synthases. J Mol Biol 328:335–363. https://doi.org/10.1016/S0022-2836(03)00232-8) and Keatinge-Clay and coworkers (Keatinge-Clay AT, Shelat AA, Savage DF, Tsai SC, Miercke LJW. O’Connell D, 3rd, Khosla C, Stroud RM. 2003. Catalysis, Specificity, and ACP Docking Site of Streptomyces coelicolor Malonyl-CoA:ACP Transacylase. Structure 11:147–154. https://doi.org/10.1016/s0969-2126(03)00004-2), are shown. Leucine and isoleucine residues typically found in AT domains selective for methylmalonyl-CoA are shown in pink (Keatinge-Clay AT, Shelat AA, Savage DF, Tsai SC, Miercke LJW, O’Connell JD, 3rd, Khosla C, Stroud RM. 2003. Catalysis, Specificity, and ACP Docking Site of Streptomyces coelicolor Malonyl-CoA:ACP Transacylase. Structure 11:147–154. https://doi.org/10.1016/s0969-2126(03)00004-2). Protein GenBank accession numbers are also shown. Download FIG S6, DOCX file, 0.03 MB. Copyright © 2022 Matilla et al. 2022 Matilla et al. https://creativecommons.org/licenses/by/4.0/ This content is distributed under the terms of the Creative Commons Attribution 4.0 International license.

**FIG 5:**
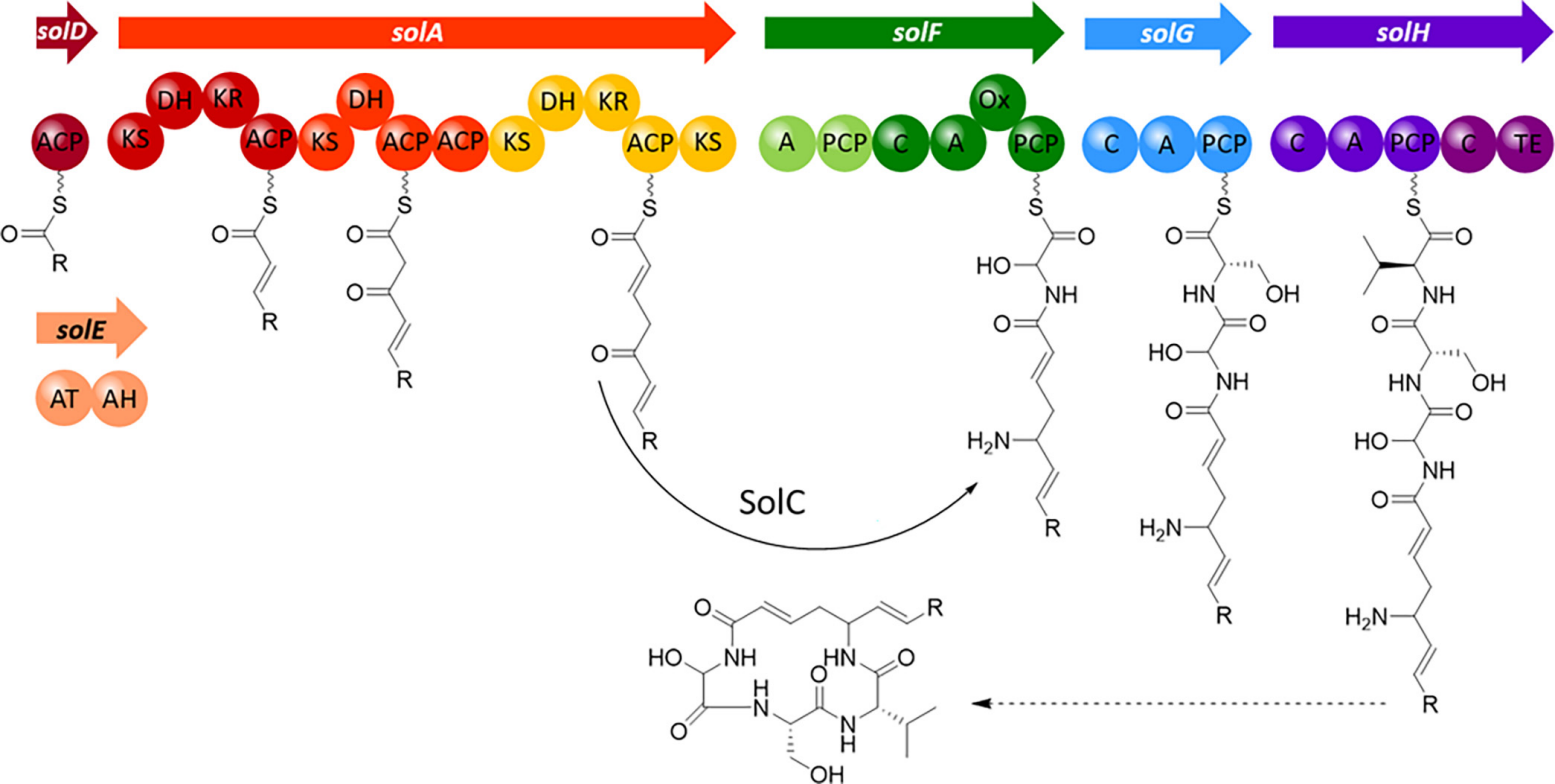
Model for the structure of the first enzyme-free precursor of solanimycin. The proposed structure is a cyclic tetrapeptide incorporating a PKS-synthesized δ-amino acid and three proteinogenic α-amino acids (Gly-Ser-Val, with the Gly residue oxidized). In this model, it was assumed that the PKS/NRPS proteins are involved in the biosynthesis in the same order as they are found in the genome. This compound would most probably be further modified by further “tailoring” enzymes in the cluster (e.g., SolB, SolC, SolI, SolJ, and SolK). ACP, acyl carrier protein; KS, ketosynthase; DH, dehydratase; KR, ketoreductase; C, condensation; A, adenylation; PCP, peptidyl carrier protein; ox, oxidase; TE, thioesterase; AT, acyltransferase; and AH, acyl hydrolase.

The in-frame deletion of the genes encoding the putative tailoring enzymes SolB, SolC, SolI, SolJ, and SolK resulted in dissimilar phenotypes. Mutants lacking *solB*, *solC*, or *solI* no longer exhibited antifungal activity, whereas a deletion mutant defective in the putative dehydratase SolK resulted in reduced antifungal activity (Fig. 4). Conversely, the in-frame deletion of putative hydrolase-encoding gene *solJ* did not alter the bioactive properties of MK10-OocN (Fig. 4). Given that *solJ* is absent in several biosynthetic clusters (Fig. 2, Fig. 3, and Fig. S2), we hypothesize that this hydrolase may have a secondary role for the final biological activity of solanimycin. Interestingly, the deletion of *solB*, *solI*, or *solK* caused the emergence of a strong orange pigmentation when these mutant strains were grown on PD medium (Fig. 4A). Unexpectedly, given the level of conservation within the various biosynthetic clusters (Fig. 2, Fig. 3, and Fig. S2), the deletion of transporter-encoding gene *solL* did not affect the antagonist properties of MK10-OocN (Fig. 4), suggesting that an alternative secretion system may be encoded in the genome of MK10. Based on the most common genetic organization of the *sol* cluster (e.g., MK10 organization) (Fig. 1 to 3 and Fig. S2), we have proposed a model for the biosynthesis of solanimycin (Fig. 5).

### Solanimycin production is regulated by RsmA and two quorum-sensing systems.

**FIG 6:**
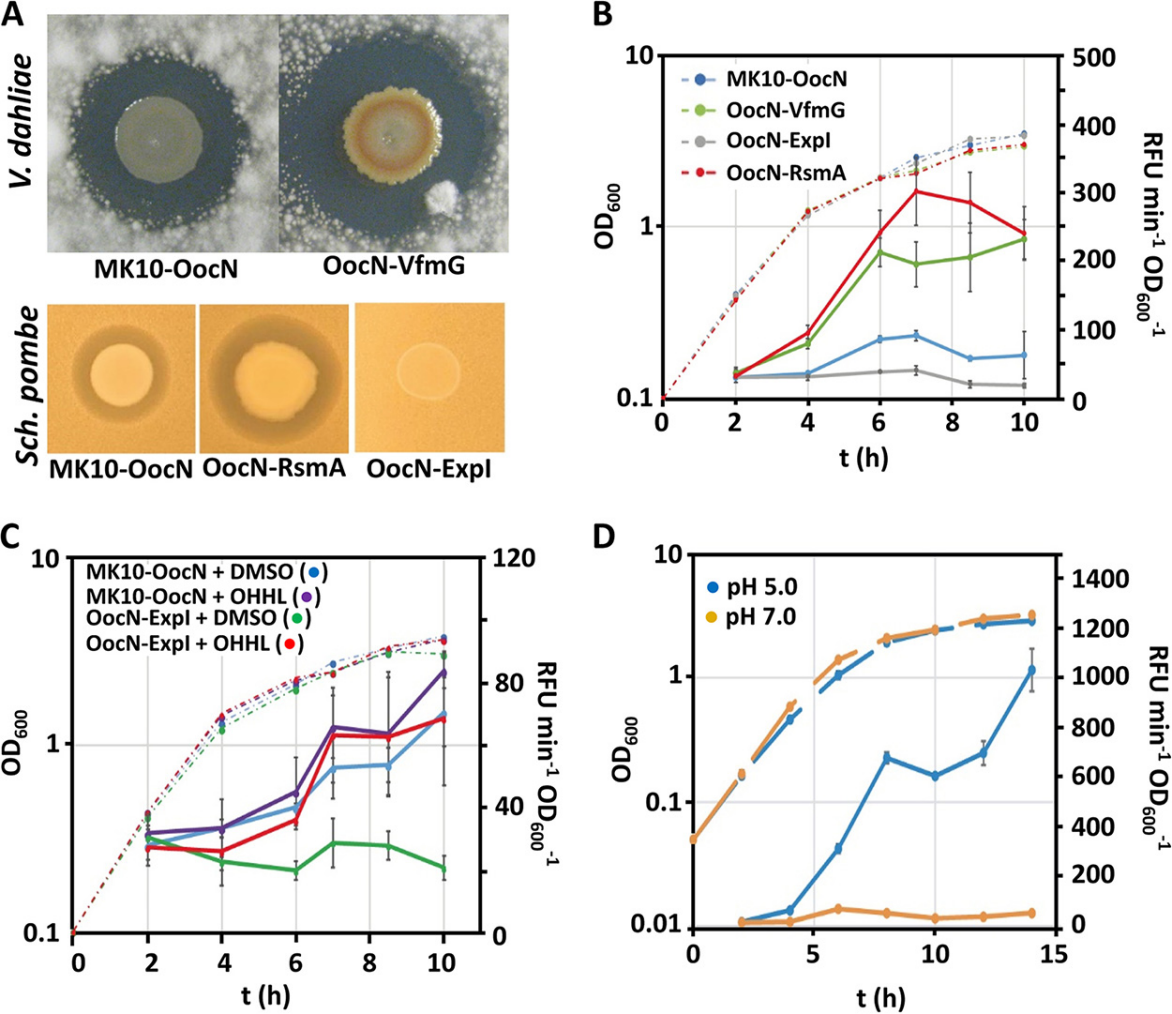
RsmA, two quorum-sensing systems, and pH modulate solanimycin production in Dickeya solani MK10. (A) Antifungal activity of mutants defective in *expI*, *rsmA*, and *vfmG* against Verticillium dahliae and Schizosaccharomyces pombe. The size of the inhibition halos is indicative of the susceptibility of the tested fungi, which are present in the top agar lawns, to solanimycin. Genotypic characteristics of the strains used are detailed in Table S1 in the supplemental material. (B) β-Galactosidase activity (solid lines) throughout growth (dashed lines) in PD medium at 25°C measured from a chromosomal fusion *solA*::*lacZ* in different MK10 genetic backgrounds. (C) Expression of the *sol* gene cluster (*solA*::*lacZ*) in different genetic backgrounds, as determined by β-galactosidase activity (solid lines), throughout growth (dashed lines) in PD medium at 25°C with and without the addition of 1 μM OHHL. (D) Expression (solid lines) of the *sol* gene cluster (*solA*::*lacZ*) in PD medium at different pH levels. Dashed lines represent bacterial growth. In panels B to D, data are the mean and standard deviation of three biological replicates.

To learn more about the regulation of solanimycin production by MK10, a random transposon mutant library of over 20,000 transconjugants was screened for altered solanimycin production. Mutants showing increased solanimycin production contained transposon insertions in the ORF encoding VfmG, a part of the signal export in the Vfm quorum-sensing (QS) system (40, 41), or in the ORF encoding RsmA, a widely studied post-transcriptional regulator of secondary metabolism (42, 43) (Fig. 6A). In addition, as part of a simultaneous screen for other QS mutants, we identified a transposon insertion in the acyl-homoserine lactone (AHL) synthase encoding gene *expI* that had no solanimycin production (Fig. 6A). ExpI is responsible for the production of two AHL-signaling molecules, *N*-(3-oxohexanoyl)-l-homoserine lactone (OHHL), the dominant molecule, and small amounts of *N*-hexanoyl-l-homoserine (44). In accordance with this, solanimycin production was restored when the *expI* gene was provided in *trans* or by addition of 1 μM OHHL to the culture medium (Fig. S5B).

To further study each of the regulatory mutants in *vfmG*, *rsmA*, and *expI*, transposon insertions were transduced into MK10-OocN or into a strain carrying a *solA*::*lacZ* transcriptional fusion for further examination throughout growth. In a *vfmG* mutant, an enhanced solanimycin production, which was detected earlier in the growth phase, was observed. Thus, solanimycin activity was detected in MK10-OocN after 14 h of growth, as the culture transitioned into stationary phase, whereas in a *vfmG* mutant, solanimycin activity was observed in culture supernatants after 6 h of growth when the culture was still growing exponentially (Fig. S7A). Similarly, in an *rsmA* mutant, solanimycin production was observed earlier in the growth phase (Fig. S7A). To determine whether the impact of each of the corresponding mutations was on the transcription of the biosynthetic cluster, we examined the expression of a *solA*::*lacZ* transcriptional fusion in conjunction with mutations in *vfmG*, *rsmA*, or *expI*. In both *vfmG* or *rsmA* mutants, β-galactosidase activity was significantly earlier at the growth phase and produced at elevated levels compared with the wild-type MK10 (Fig. 6B). We concluded therefore that the posttranscriptional regulator RsmA and the Vfm QS system repress transcription of the *sol* biosynthetic cluster. Additionally, no appreciable *solA* transcription was observed at all time points in an *expI* mutant, and we could restore *solA* transcription by the addition of 1 μM OHHL to the medium (Fig. 6C). We also noted that transcription of the *sol* cluster was not precociously induced on addition of OHHL (Fig. 6C), suggesting that the ExpIR system acts within a wider regulatory network to control solanimycin production.

10.1128/mbio.02472-22.8 FIG S7 Influence of growth conditions in solanimycin production in Dickeya solani MK10 strains. (A) Solanimycin production by MK10 strains throughout growth in PD medium. Solid lines represent halo area/OD_600_, whereas dashed lines represent bacterial growth. (B) Solanimycin production by D. solani MK10-OocN in buffered and unbuffered PD medium. For the assays in panels A and B, an S. pombe top agar lawn was prepared, and 300 μL of filter-sterilized supernatants was added to holes punched in the S. pombe plates. (C) Changes throughout growth in the pH of YES medium by yeasts. Data are the mean and standard deviation of three biological replicates. (D) Effect of buffering YES medium on solanimycin production. Halos of antibiosis against S. pombe of filter-sterilized supernatants of D. solani MK10-OocN grown in PD and YES medium buffered at different pH levels. In all cases, the bioassays were repeated at least three times, and representative results are shown. Download FIG S7, JPG file, 0.5 MB. Copyright © 2022 Matilla et al. 2022 Matilla et al. https://creativecommons.org/licenses/by/4.0/ This content is distributed under the terms of the Creative Commons Attribution 4.0 International license.

### Production of solanimycin is enhanced under conditions that mimic the plant host environment.

**FIG 7:**
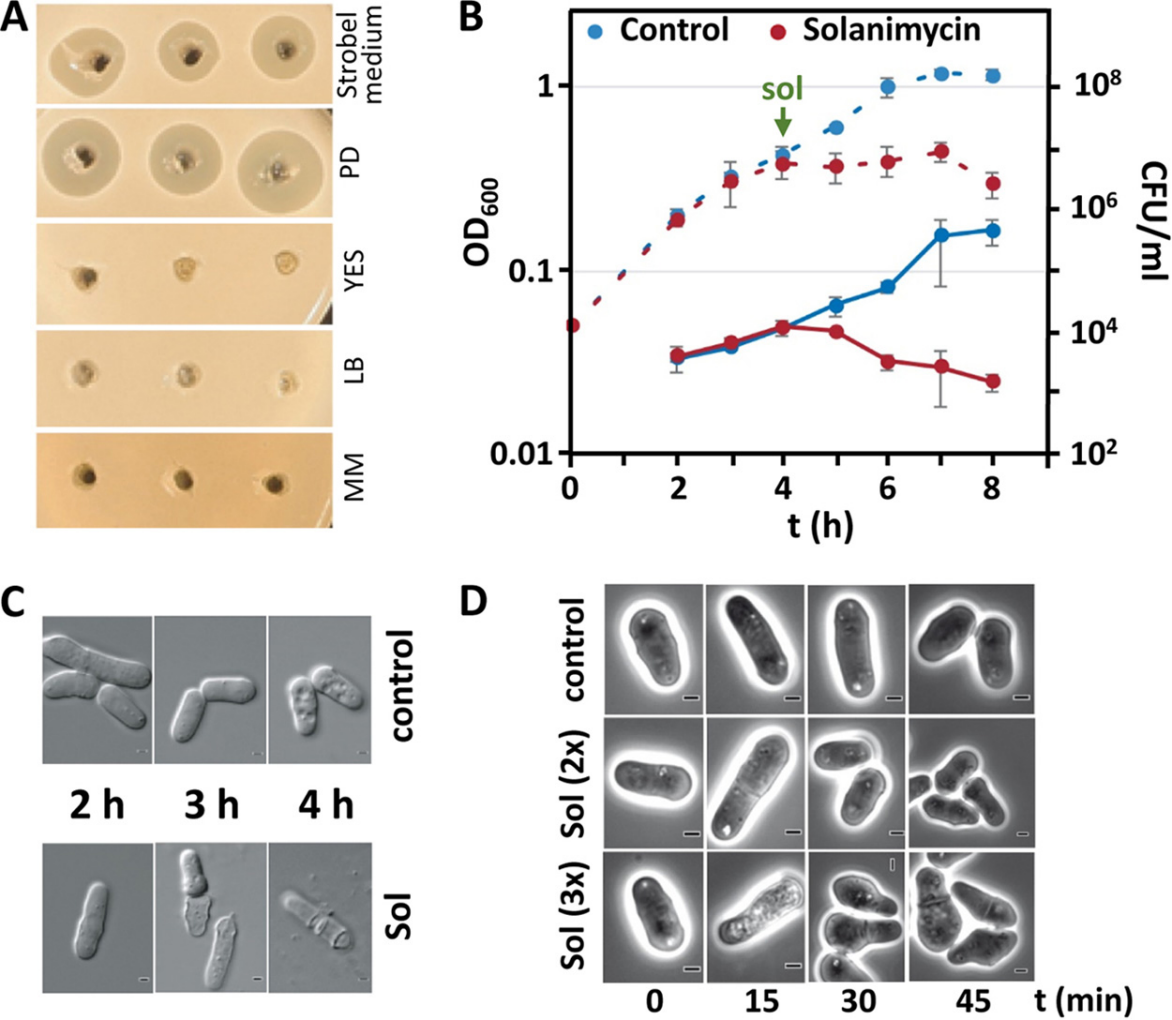
Purified solanimycin impacts growth and development of S. pombe. (A) Growth inhibition of S. pombe with culture supernatants of D. solani MK10 in Strobel, potato dextrose (PD), YES, LB, and minimal medium (MM). (B) S. pombe growth (dashed lines, OD_600_) with partially purified solanimycin (sol; concentration 0.6× that of an original MK10 wild-type culture) added after 4 h of growth. The solvent (40% ethanol) used to dissolve partially purified solanimycin was used as a control. Average colony counts were assessed throughout growth (solid lines). Data are the mean and standard deviation of three biological replicates. (C) Representative microscopy images of S. pombe cells with either partially purified solanimycin at 0.6× or the solvent control from cultures after different times after addition. (D) S. pombe cells grown in higher concentrations of partially purified solanimycin (2× and 3×, as indicated) and imaged immediately (0 min) and at different time points after solanimycin addition. All images are representative of those observed. Scale bars, 1 μm.

We found that solanimycin production was dependent on the culture medium. Activity was detected in culture supernatants late in stationary phase when grown in PD or Strobel medium, but no activity was detected in culture supernatants at any time throughout growth in LB, YES, or minimal medium (Fig. 7A). Acidic pH is a stress faced by *Dickeya* spp. during early stages of plant infection, and low pH has been shown to modulate virulence in different *Dickeya* spp. (45, 46), including species that contain the *sol* biosynthetic cluster. High levels of solanimycin production were observed in PD (Fig. 7A), a culture medium that mimics the pH and nutrients present in potato tubers. The pH of the PD medium before inoculation was 5.1 and after 12 h of MK10 growth fell to 4.2. To examine the effect of pH on solanimycin production further, PD medium buffered to either pH 7.0, 6.0, or 5.0 (D. solani failed to grow at pH 4.0) was inoculated with MK10-OocN and solanimycin production monitored. Antifungal activity was only observed in supernatants from cultures grown at pH 5.0 (Fig. S7B). We also examined transcriptional activity of the *sol* cluster at different pH levels, and a 200-fold increase in *solA* transcription was observed between media buffered to pH 5.0 and pH 7.0 (Fig. 6D), indicating the key role of pH as a regulatory input for solanimycin biosynthesis. In accordance, we found that Saccharomyces cerevisiae or Schizosaccharomyces pombe, in the absence of D. solani, lowers the pH of the YES broth, a medium that does not promote solanimycin synthesis, ultimately settling at a pH of approximately 4.4 (Fig. S7C). We buffered the YES medium to pH 7.0, 6.0, and 5.0 and cultured MK10-OocN under these conditions. Solanimycin production was only detected in cultures grown at pH 5.0 (Fig. S7D), suggesting that the acidic conditions created by high-density yeast cultures helped to induce solanimycin production.

### Biological properties of solanimycin and its impact on eukaryotic cell viability.

**FIG 8:**
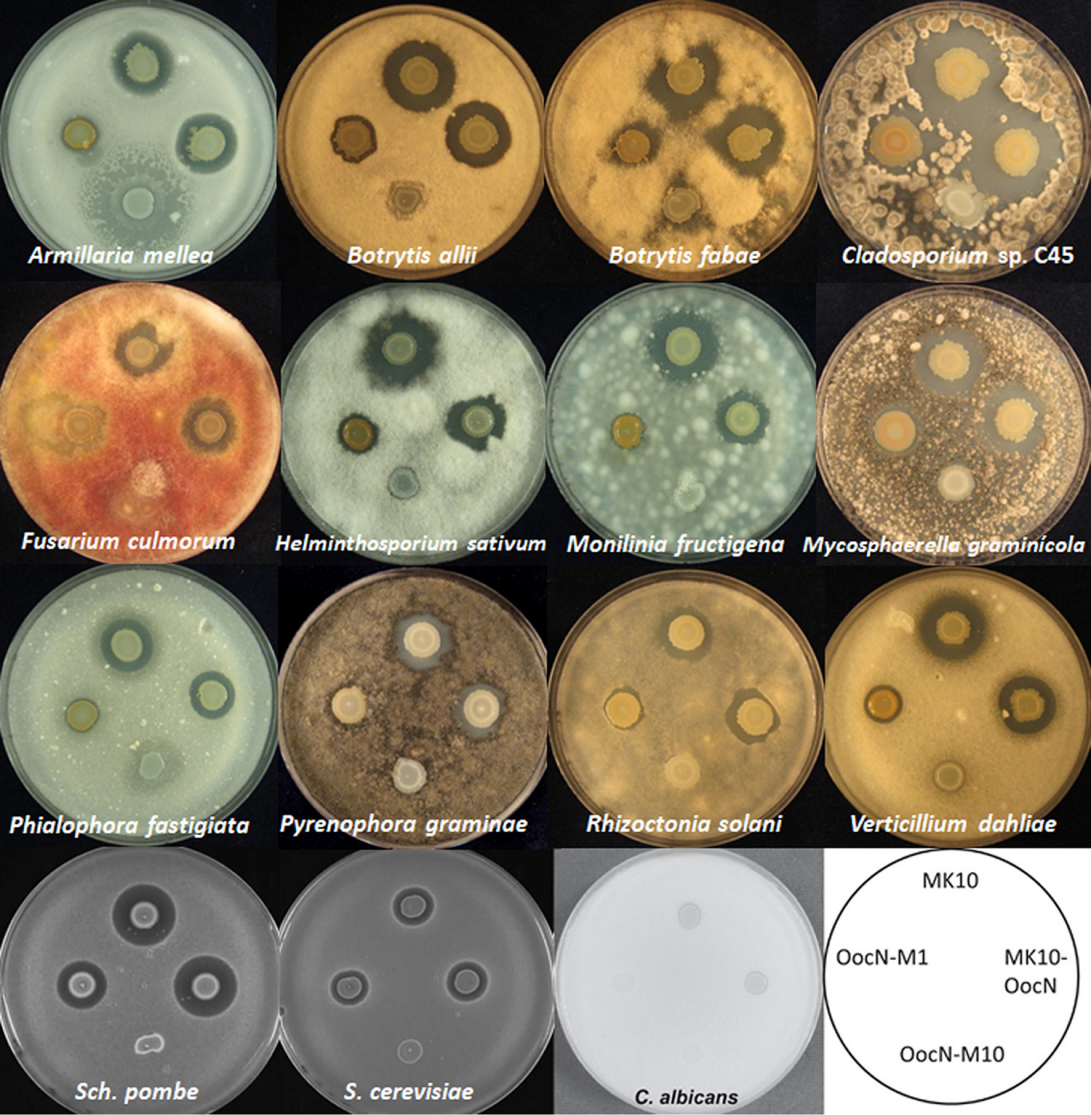
Broad range of antifungal properties of solanimycin. Bioactivities of D. solani MK10 and derivative strains defective in the synthesis of oocydin A (MK10-OocN) and both oocydin A and solanimycin (OocN-M10 and OocN-M1). The size of the inhibition halos is indicative of the susceptibility of the tested fungi, which are present in the top agar lawns, to oocydin A and solanimycin. Genotypic characteristics of the bacterial strains used are detailed in Table S1 in the supplemental material. The bioassays were repeated at least three times, and representative pictures are shown. Pictures were taken after 3 to 7 days of incubation at 25°C.

We phenotypically compared MK10 and mutant derivatives defective in the synthesis of oocydin A (MK10-OocN) and both oocydin A and solanimycin (OocN-M10 and OocN-M1). First, we assessed the antagonistic activities toward 26 plant-pathogenic fungi of the *Ascomycota* and *Basidiomycota* phyla, including fungi belonging to 5 different classes and 12 orders. These fungi included phytopathogens ranked in the top 10 in plant pathology (e.g., Botrytis cinerea and Fusarium oxysporum) (47), as well as a number of fungal pathogens of potato (e.g., Colletotrichum coccodes, Fusarium solani, Rhizoctonia solani, and Verticillium dahliae). We observed that solanimycin was active against around 70% of the tested plant-pathogenic fungi (Fig. 8 and Fig. S8). Among the most susceptible fungi, we identified economically important phytopathogens such as Armillaria mellea, Botrytis allii, Botrytis cinerea, Botrytis fabae, Fusarium culmorum, Helminthosporium sativum, Monilinia fructigena, Mycosphaerella graminicola, Pyrenophora graminea, and Rhizoctonia solani (Fig. 8 and Fig. S8). We also found that solanimycin was active against the ascomycete yeasts Candida albicans, Saccharomyces cerevisiae, and Schizosaccharomyces pombe (Fig. 8). No activity against various Gram-positive or Gram-negative bacteria was detected, and there was no obvious association of solanimycin with bacterial virulence in Caenorhabditis elegans models (Fig. S1E), implying that solanimycin may be specifically targeting members of the fungal kingdom.

10.1128/mbio.02472-22.9 FIG S8 Antifungal properties of solanimycin. Bioactivities of D. solani MK10 and derivative strains defective in the synthesis of oocydin A (MK10-OocN) and both oocydin A and solanimycin (OocN-M10 and OocN-M1). The size of the inhibition halos is indicative of the susceptibility of the tested fungi, which are present in the top agar lawns, to solanimycin. This figure also includes bioassays in which no solanimycin-mediated antagonistic activity was observed (e.g., Corticium solani, Penicillium crustosum, Rhizoctonia cerealis, and Rhizoctonia oryzae). We have decided to keep these bioassays to show the absence of activity against these phytopathogens. Genotypic characteristics of the strains used are detailed in Table S1. The bioassays were repeated at least three times, and representative pictures are shown. Pictures were taken after 3 to 7 days of incubation at 25°C. Download FIG S8, JPG file, 1.4 MB. Copyright © 2022 Matilla et al. 2022 Matilla et al. https://creativecommons.org/licenses/by/4.0/ This content is distributed under the terms of the Creative Commons Attribution 4.0 International license.

We addressed solanimycin detection in cell-free culture supernatants using an S. pombe cut-well assay. Solanimycin was partially purified from supernatants of cultures grown in PD medium, and the fractions containing antifungal activity were examined by ultraperformance liquid chromatography-quadrupole time of flight mass spectrometry (UHPLC-Q-TOF MS) analysis. Two metabolites were identified in the wild-type supernatant fraction but not in the OocN-M10 fraction (Fig. S9). These had *m/z* values of 963.48 and 979.474, a difference in molecular weight corresponding to one oxygen. This partially purified compound was added to cultures of actively growing S. pombe. Within 1 h of exposure, a growth inhibition was observed, and viable colony counts showed a significant decrease compared with the solvent control. Three hours after solanimycin addition, a 542-fold reduction in viable colonies was observed, suggesting that the action of solanimycin was rapid (Fig. 7B). In addition, differences in cell morphology and dead cells were observed (Fig. 7C). We also examined S. pombe cells grown with higher concentrations of solanimycin, namely, two and three times the concentration found in *Dickeya* supernatants. Dead cells were visible at these higher concentrations after 15 to 30 min, and morphological changes, such as rounded-off cells, incorrectly placed division septa, stress granules, and aggregation, were observed (Fig. 7D), indicating that the impacts of solanimycin on eukaryotic yeast cells were quick and lethal.

10.1128/mbio.02472-22.10 FIG S9 Two ions are associated with purified solanimycin activity. Triplicate LC-MS traces of MK10-OocN (top traces in each panel) or OocN-M10 (bottom three traces in each panel) partially purified culture supernatants. (A) Extracted ion chromatograph for ion 963.48; (B) extracted ion chromatograph for ion 979.474. Download FIG S9, JPG file, 1.4 MB. Copyright © 2022 Matilla et al. 2022 Matilla et al. https://creativecommons.org/licenses/by/4.0/ This content is distributed under the terms of the Creative Commons Attribution 4.0 International license.

## DISCUSSION

Dickeya solani (30) was first reported in European seed potato stocks from 2005 to 2006, and it is now recognized as a prominent plant pathogen worldwide (48, 49). This success may be associated with the organism’s ability to colonize plant tissues rapidly and overwhelm competitors (29, 34, 50), for example, through the production of an array of bioactive secondary metabolites that, among their ecological functions, act as intermicrobial warfare agents in the killing or inhibition of competitors (51–53). In accordance, recent genomic analyses proposed that the success of D. solani strains may be due to the presence and combination of NRPS/PKS clusters (28–30, 54). Indeed, the divergence of D. solani from other *Dickeya* spp. has been linked with the acquisition of additional secondary metabolite clusters by D. solani (30). Specifically, D. solani strains were shown to produce the antibacterial, antifungal, and nematicide hybrid NRP/PK zeamine (31), the antifungal and antioomycete PK oocydin A (34), and, as reported here, the antifungal hybrid NRP/PK solanimycin.

The *sol* biosynthetic cluster was found in many *Dickeya* spp., as well as in other phylogenetically distant bacterial genera (Fig. 2 and Fig. S2). While the cluster seemed to be most prevalent in the genomes of *Dickeya* spp., this may reflect a bias toward genome sequencing of economically important agricultural pathogens rather than commensal or saprophytic microbes isolated from the same habitats. An interesting finding from this work was the different configurations of the putative tailoring enzymes within the *sol* cluster (Fig. 3) and the loss or addition of NRPS modules in SolF/H encoded within the clusters of different strains (Fig. 2 and Fig. S2). These differences could help drive chemical diversity from each genomic configuration, which has been reported in other bacteria. For example, the daptomycin and glycopeptidolipid biosynthetic clusters from Streptomyces roseosporus and Mycobacterium avium, respectively, are genetically similar, but their final products differ in structure (55–57). Also, it was found that the HGT of different tailoring genes contributes to the structural and biosynthetic diversity of pentangular polyphenol polyketides (58). Currently, we are investigating the chemical structure of solanimycin and beginning to investigate possible links between genomic diversity and potential chemical diversity elaborated from the *sol* loci.

The biosynthesis of secondary metabolites can be energetically demanding, and therefore, their production is often tightly regulated by different chemical signals and environmental cues (51, 59, 60) such that many secondary metabolite clusters can be phenotypically cryptic under standard growth conditions (61–63). Various secondary metabolites are known to be under AHL QS control (64–66), but the specific role of the ExpIR system in D. solani remains unclear. In some D. solani isolates, an *expI* mutant shows reduced potato maceration (67). However, in several D. solani strains, the production levels of major virulence determinants, such as secreted pectate lyase and cellulase, in *expI* mutants were indistinguishable from the corresponding wild-type strain (40, 67). Here, we found that solanimycin was regulated in response to OHHL, suggesting that the local presence of other OHHL producers, for example, alternative soft-rotting bacteria (68, 69), might induce solanimycin biosynthesis during mixed infection of the plant. However, the regulatory hierarchy controlling solanimycin production was more complicated, with multiple regulatory inputs, including the recently discovered Vfm QS system (40, 41, 70, 71).

Recent studies found that the Vfm system modulates motility, plant cell wall-degrading enzyme synthesis, the expression of the type VI secretion system, and plant virulence in different *Dickeya* species (70, 71). The Vfm genetic cluster is comprised of four operons across 30 kb in MK10 (72), and the *vfmG* mutants that we isolated were potentially defective for *vfmHIJ* expression due to polar effects of transposon insertion. VfmG is predicted to encode part of the Vfm signal export system, whereas VfmJ encodes a 4′ phosphopantetheinyl transferase proposed to be involved in the Vfm signal synthesis (41). Additionally, VfmH-VfmI form a classical two-component system (41, 71), and mutants defective in *vfmH* in D. dadantii and D. zeae do not produce or respond to the Vfm signaling molecule (41, 71). Thus, one explanation for the observed increased solanimycin production in a *vfmG* mutant could be the lack of signaling molecule biosynthesis in this strain, therefore representing an example of QS-based repression of a secondary metabolite (41, 71). Analogously, mutation of *rsmA* resulted in an increase in the solanimycin biosynthesis (Fig. 6A), and work conducted in different *Pectobacterium* strains revealed that the Rsm system modulates the production of QS signaling molecules (73). Our work thus suggests a complex interplay between the Vfm and ExpIR QS systems and with the Rsm regulatory pathway on solanimycin production.

Microbial communities are heterogeneous in nature and are constantly adapting to local environments. For example, the synthesis of hybrid NRP/PK antibiotic andrimid was inhibited in a root-associated bacterium by the production of indole-3-acetic acid by microbial competitors (74). Remarkably, interkingdom communication between fungi and bacteria has been also shown to strongly modulate secondary metabolite biosynthesis in the interacting partners (75). In accordance with these data, we observed that the solanimycin production in D. solani was induced when exposed to ascomycete yeasts in response to the acidic conditions, suggesting that D. solani adapts to acidic conditions mirroring potato tissues by modulating antibiotic production (76). Sensing and responding to environmental pH is an important regulatory cue used by bacteria to modulate gene expression (77). For example, acidic pH is a requirement for Salmonella enterica virulence through activation of the PhoP/PhoQ two-component system, and neutralization of the pH in macrophage phagosomes prevents S. enterica replication (78). Remarkably, D. solani regulates twitching motility in response to acidic conditions characteristic of potato tubers, and this study found that pH modulated solanimycin biosynthesis (46).

The need for new antifungals has heightened as existing medicines become less effective and new invasive fungal species emerge (9, 79–81). Furthermore, the rise in fungal infections of agricultural crops, plant ecosystems, and livestock provides a further incentive to identify new antifungal molecules (3, 9). Solanimycin displays broad activity against many important human- and plant-pathogenic fungi. From a pharmaceutical and agricultural perspective, solanimycin may represent a potentially exciting discovery, and this study confirms the view that soil- and plant-associated microbes other than actinomycetes represent an underexplored reservoir of bioactive secondary metabolites with potential medicinal and agricultural utility. Here, we showed that solanimycin production is highly regulated at several levels. The understanding of these regulatory mechanisms will not only advance our knowledge of the ecological function of this antibiotic but will also lay the foundation for its applied use. For example, the heterologous expression of the antibiotic biosynthetic clusters in safe biocontrol strains has been suggested as a strategy for the development of new-generation biopesticides (82).

## MATERIALS AND METHODS

### Strains, plasmids, oligonucleotides, and culture conditions.

Bacteria, fungi, oomycete, and phages used in this study are described in Table S1 in the supplemental material. Plasmids and oligonucleotides are listed in Table S1. *Dickeya* strains were routinely grown at 25°C, unless otherwise indicated, in Luria broth (LB), potato dextrose (PD), Strobel medium (83), or minimal medium (34). Escherichia coli strains were grown at 37°C in LB. E. coli DH5α was used as a host for gene cloning. When appropriate, antibiotics were used at the following final concentrations (in μg mL^−1^): ampicillin, 100; kanamycin, 50; and streptomycin, 50. Sucrose was added to a final concentration of 10% (wt/vol) to select derivatives that had undergone a second crossover event during marker exchange mutagenesis. Media for propagation of E. coli β2163 were supplemented with 300 μM 2,6-diaminopimelic acid. S. cerevisiae, S. pombe, and C. albicans were grown at 30°C in yeast peptone dextrose (YPD) or in yeast extract with supplements (YES).

### *In vitro* nucleic acid techniques and bioinformatic analyses.

Genomic and plasmid DNA was isolated using the Thermo Fisher GeneJet extraction kits. Phusion high-fidelity DNA polymerase (New England Biolabs) was used in the amplification of PCR fragments for cloning. PCRs and DNA fragments were purified or recovered from agarose using Thermo Fisher’s PCR clean-up and gel purification kits, respectively. Restriction (New England Biolabs) and ligation (Thermo Fisher T4 DNA ligase) reactions were performed according to manufacturers’ instructions. Genome comparison analyses were performed employing wgVISTA online tool (84), whereas protein domain organization and the analysis of biosynthetic clusters were undertaken using antiSMASH v6.0 (11). ORFs were predicted using Glimmer 3.0 (85). Multiple-sequence alignments were carried out with ClustalW2 (European Bioinformatics Institute).

### Random transposon mutagenesis.

Random transposon mutagenesis was performed using mini-Tn*5-*Sm/Sp or the plasposons pKRCPN1 and pDS1028 by biparental conjugation mating, as described previously (36). Random mutants were screened for their antifungal activity against Verticillium dahliae, S. cerevisiae, and S. pombe using dual drop culture bioassays or for lack of β-galactosidase activity on an LBA plate containing 40 μg mL^−1^ 5-bromo-4-chloro-3-indoyl-β-d-galactopyranoside. In the case of the *expI* mutant, loss of violacein production was tested on a Chromobacterium violaceum CV026 top lawn (86). Auxotrophic mutants were discarded, and insertion mutations were transduced into D. solani strains using phage ϕXF1 (87). The transposon insertion sites were determined using random primed PCR following the method described previously (36, 88) and using oligonucleotides described in Table S1.

### Construction of in-frame deletions and complementation plasmids.

The plasmids for the construction of the in-frame deletions mutants were generated by amplifying the up- and downstream flanking regions of the gene to be deleted. The resulting PCR products were digested with the enzymes specified in Table S1 and ligated in a three-way ligation into pUC18Not or pBluescript2SK+ prior to being cloned into the marker exchange vector pKNG101. Plasmids for mutagenesis were transferred to D. solani MK10-OocN by biparental conjugation using E. coli β2163. The in-frame deletion mutant strains OocN-SolA, OocN-SolB, OocN-SolC, OocN-SolD, OocN-SolE, OocN-SolF, OocN-SolG, OocN-SolH, OocN-SolI, OocN-SolJ, OocN-SolK, and OocN-SolL were constructed by homologous recombination using plasmids pKNG101-*solA*, pMAMV219, pMAMV218, pMAMV217, pMAMV216, pKNG101-*solF*, pKNG101-*solG*, pKNG101-*solH*, pMAMV215, pMAMV214, pMAMV213, and pMAMV212, respectively (Table S1). All relevant mutations were confirmed by PCR and sequencing.

For the construction of the complementation plasmids, the relevant genes were amplified using the indicated oligonucleotides described in Table S1. The resulting PCR products were digested with the enzymes specified in Table S1 and cloned into pTA100 or pQE80-*oriT*. Complementing plasmids were used to transform *Dickeya* strains by electroporation or conjugation. Gene expression was induced by the addition of isopropyl-β-d-thiogalactopyranoside (IPTG) at 1 mM.

### Phenotypic bioassays.

Antagonistic activities against bacteria and plant-pathogenic fungi and oomycetes were assayed as described previously (35). All antagonistic assays were done in PD agar medium. For the fungicide assays, 5 μL of overnight cultures of the selected strains was spotted on the surface of a fungal agar lawn and incubated for 4 to 10 days at 25°C. For the antioomycete assays, 5 μL of overnight bacterial cultures was spotted on PD agar plates. Following incubation for 16 h at 25°C, the plates were inoculated with 5-mm-diameter mycelial plugs taken from a culture of Pythium ultimum grown on PD agar. Fungicidal activities against C. albicans, S. cerevisiae, and S. pombe were carried out in YPD or YES media. The analysis of virulence using Caenorhabditis elegans models were performed as previously described (31). In all cases, the assays were performed at 25°C. Production of acyl-homoserine lactones was assessed by examining violacein production on a C. violaceum CV026 bioassay plate (86).

### Microscopy.

Microscopy of S. pombe cells was undertaken at specific time points throughout growth and analyzed by phase-contrast microscopy (PCM) with an Olympus BX51 microscope using a QICAM monochrome camera or by differential interference contrast (DIC) microscopy using a Nikon microscope and Nikon Vision software. PCM images were processed as described previously (89), and DIC images were processed using Nikon Vision software according to the manufacturer’s setup.

### Solanimycin partial purification.

Sterile-filtered supernatant was lyophilized and extracted with methanol (~250 mL per L supernatant). After removal of residual solid material by filtration, the solvent was removed *in vacuo*. The crude extract was loaded onto LH-20 Sephadex column (40 g resin/g extract) and eluted with methanol. Fractions demonstrating antifungal activity were pooled and solvent removed *in vacuo*. This material was further purified by loading onto C_18_ resin, washing with 50% acetonitrile in water, and eluting in 70 to 100% acetonitrile.

### UHPLC/Q-TOF MS analysis.

UPLC was carried out using a Waters Acquity UPLC system on a BEH C_18_ 1.7-μm, 2.1- by 50-mm column coupled to a Waters Xevo G2-S Q-TOF MS running positive-ion mode electrospray ionization. UPLC separation was carried out with an 8-min program consisting of a 10% to 90% gradient of acetonitrile in water plus 0.1% formic acid over 7.2 min, followed by washing and reequilibration steps. An MS source temperature of 120°C, capillary voltage of 3 kV, and sampling cone voltage of 40 V were used.

### Transcriptional fusion assays.

Expression of the *sol* gene cluster was assessed by examination of a *solA*::*lacZ* transcriptional fusion, which was generated using the transposon plasmid pKRCPN1 (Table S1), which contains a promoterless *lacZ* gene and therefore permits the creation of stable transcriptional fusions in the chromosome when the Tn-KRCPN1 transposon is inserted in the same direction of the promoter be investigated. Tn-KRCPN1 insertions cause polar effect on the downstream genes. Expression of the *lacZ* reporter gene was performed using the fluorogenic substrate 4-methylumbelliferyl β-d-galactoside (Melford; catalog no. M1095) at a final concentration of 0.125 mg mL^−1^. Samples were measured in a SpectraMax Gemini XPS fluorescence microplate reader (Molecular Devices) using the following settings: excitation, 360 nm; emission, 450 nm; cutoff, 435 nm; and reading every 30 s for 20 min at 37°C. β-Galactosidase activity was expressed as relative fluorescent units per second and normalized to the optical density at 600 nm (OD_600_) of the corresponding sample. All the transcriptional fusion assays were performed using Dickeya solani MK10Lac (wild type) or mutants derived from MK10Lac.

### RNA isolation and RT-PCR.

RNA was extracted from D. solani cultures grown for 6 h in PD medium as previously described (90). Genomic DNA contamination was removed by treatment with Turbo DNase (Thermo Fisher). RNA concentrations were assessed spectrophotometrically, and reverse transcriptase reactions were performed as described previously (34). Oligonucleotides were designed to amplify across the junction between each gene in the solanimycin cluster, and those immediately flanking the cluster (Table S1) were used to assess the transcript. Positive (using MK10 genomic DNA) and negative (no RT reaction controls) were also conducted for each oligonucleotide pair.

### Bioinformatic comparisons.

Solanimycin clusters were identified using BLAST (91), and the genomic sequence and predicted proteins in the corresponding cluster were analyzed using antiSMASH (11). The GenBank files were compared using the Python-based tool clinker, and the results were visualized using clustermap.js (92). Colors were changed from the defaults to reflect common predicted protein function, and to highlight the differences in gene order, the order of the clusters was changed from the default settings.

### Data availability.

All data generated or analyzed during this study are included in this published article and its supplemental material files.
