# Supplementary figures and images for: Solanimycin: Biosynthesis and Distribution of a New Antifungal Antibiotic Regulated by Two Quorum-Sensing Systems
Source: mBio. 2022 Oct 10;13(6):e02472-22. doi: 10.1128/mbio.02472-22 (PMC9765074; doi:10.1128/mbio.02472-22)

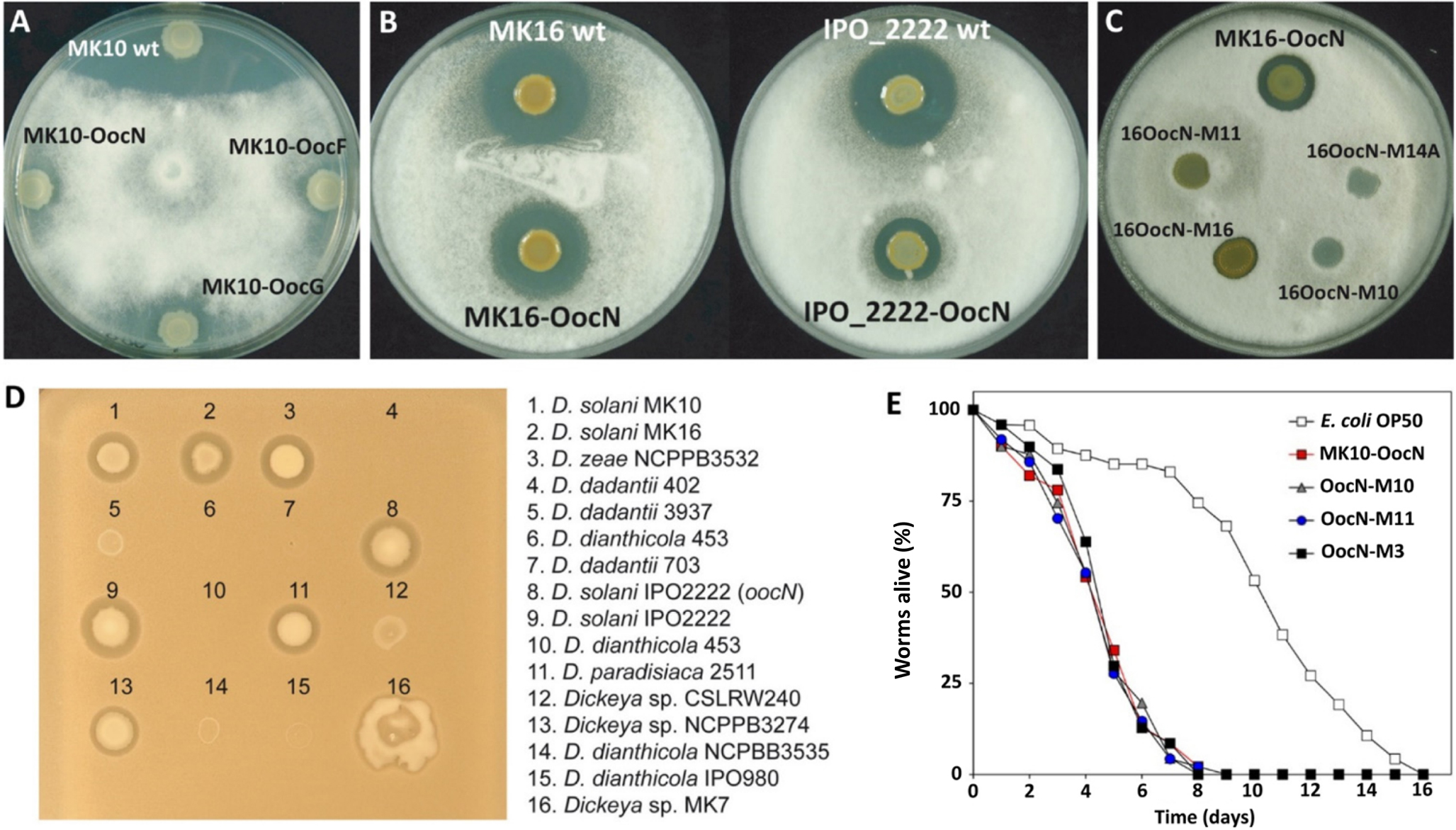

Supplement: FIG S1 [file mbio.02472-22-s0001.jpg]

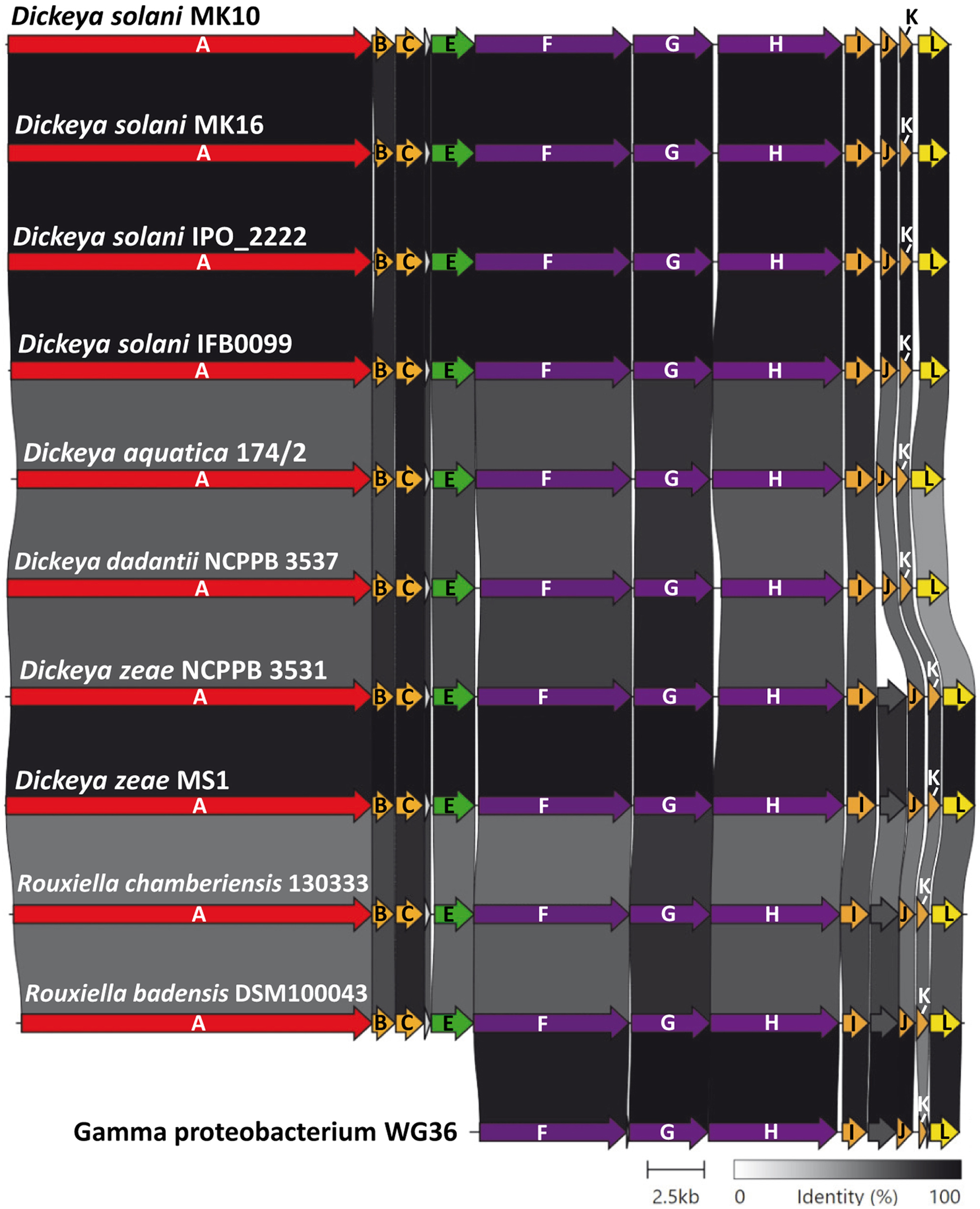

Supplement: FIG S2 [file mbio.02472-22-s0002.jpg]

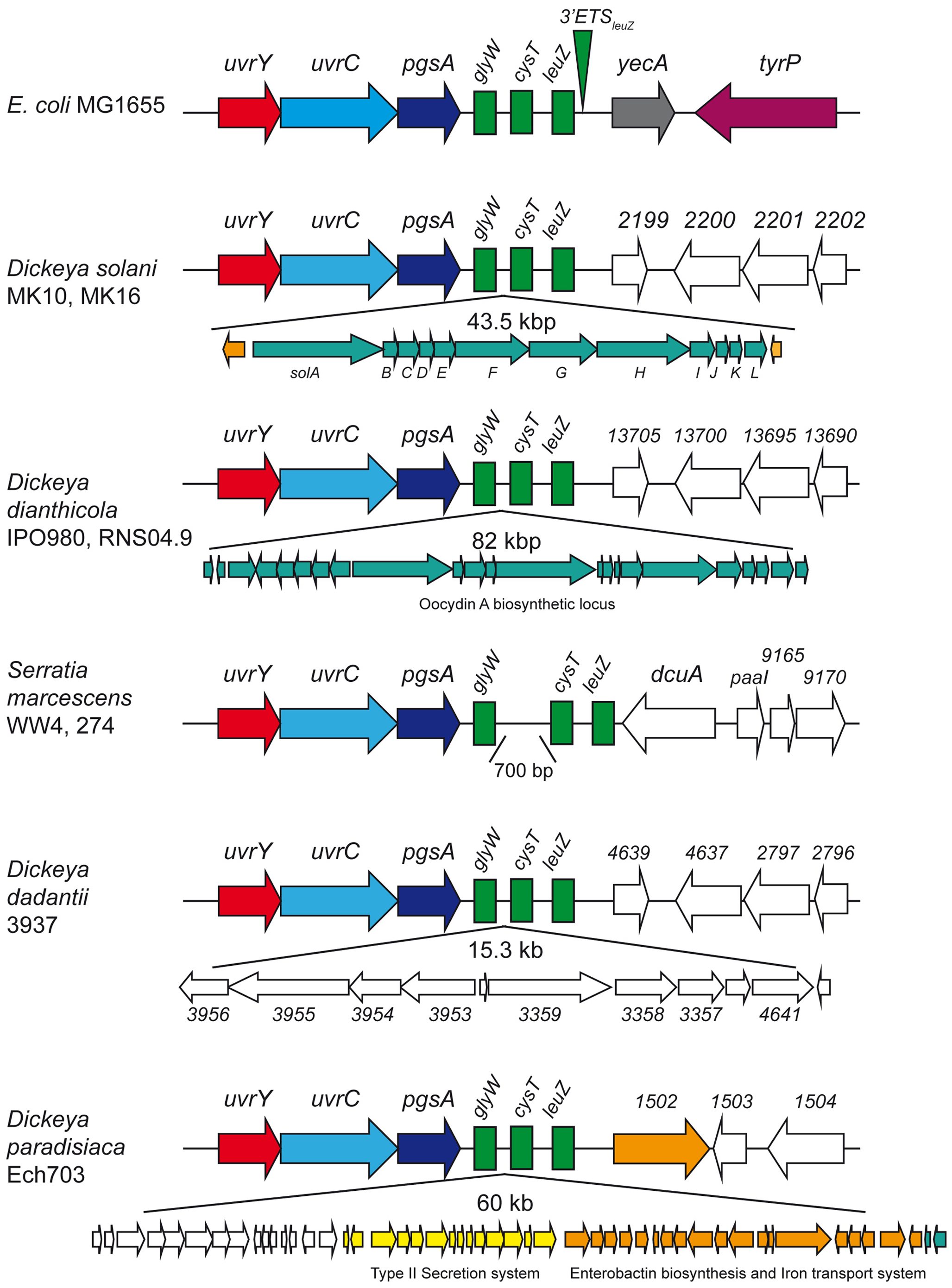

Supplement: FIG S3 [file mbio.02472-22-s0003.jpg]

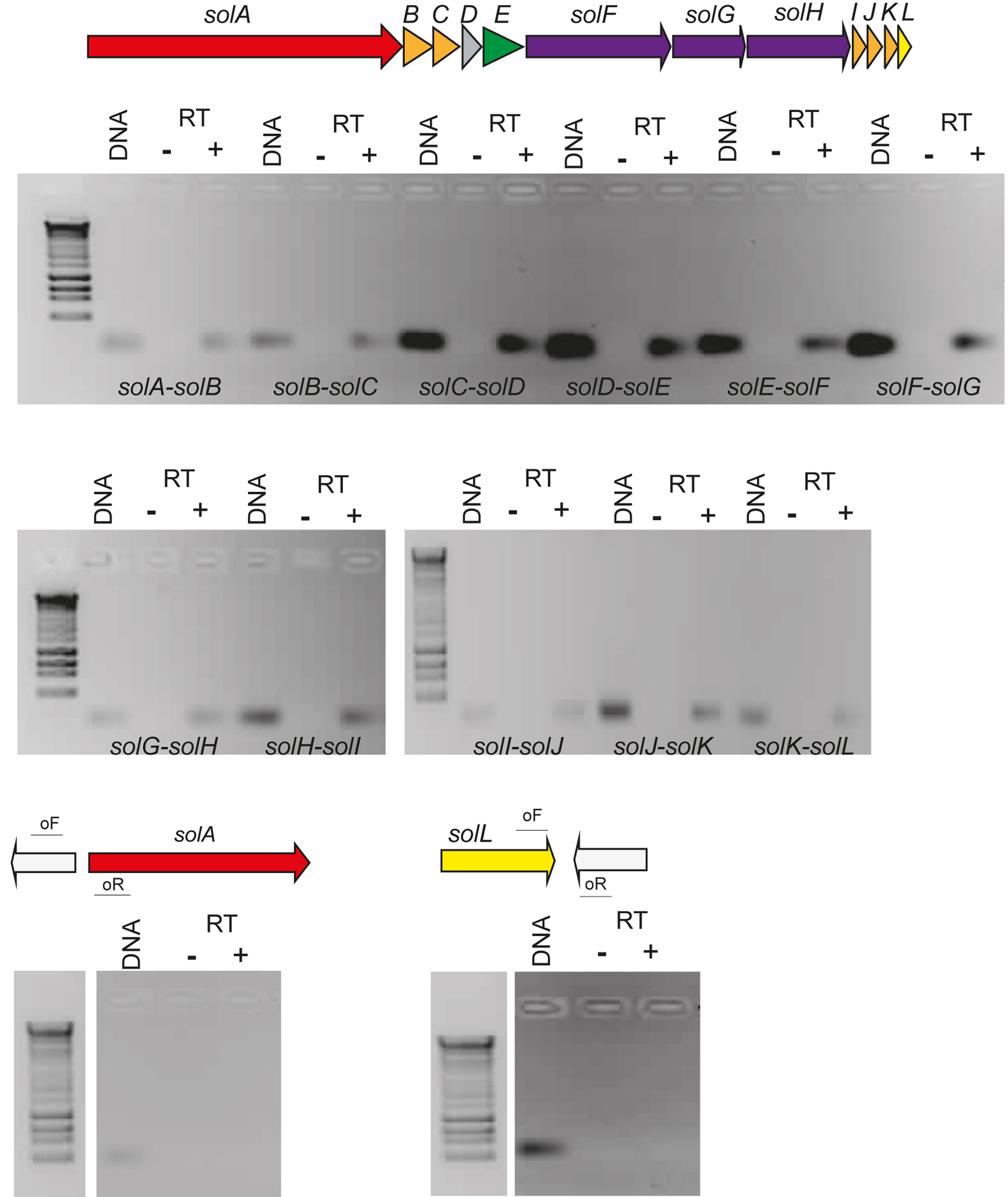

Supplement: FIG S4 [file mbio.02472-22-s0004.jpg]

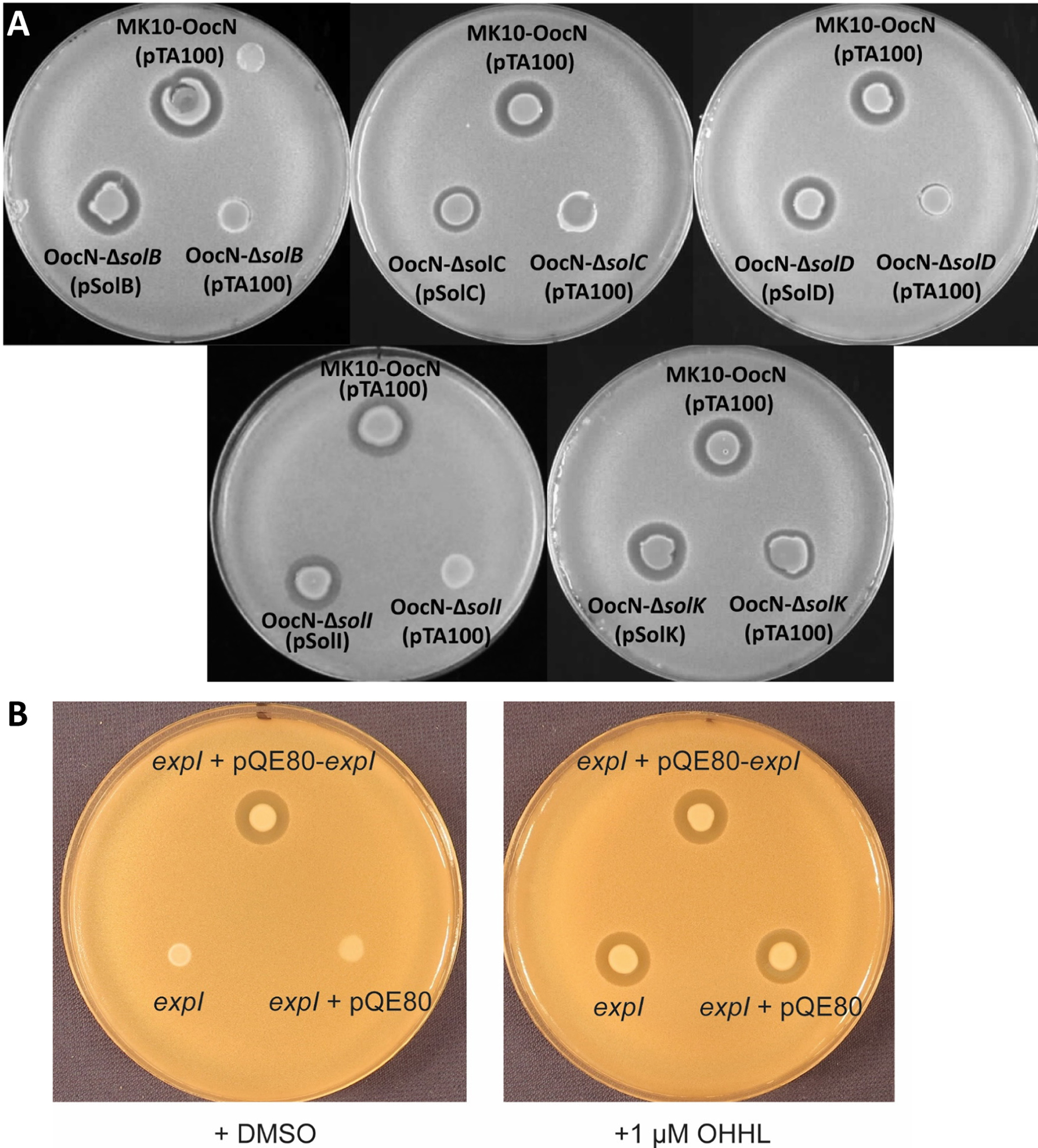

Supplement: FIG S5 [file mbio.02472-22-s0005.jpg]

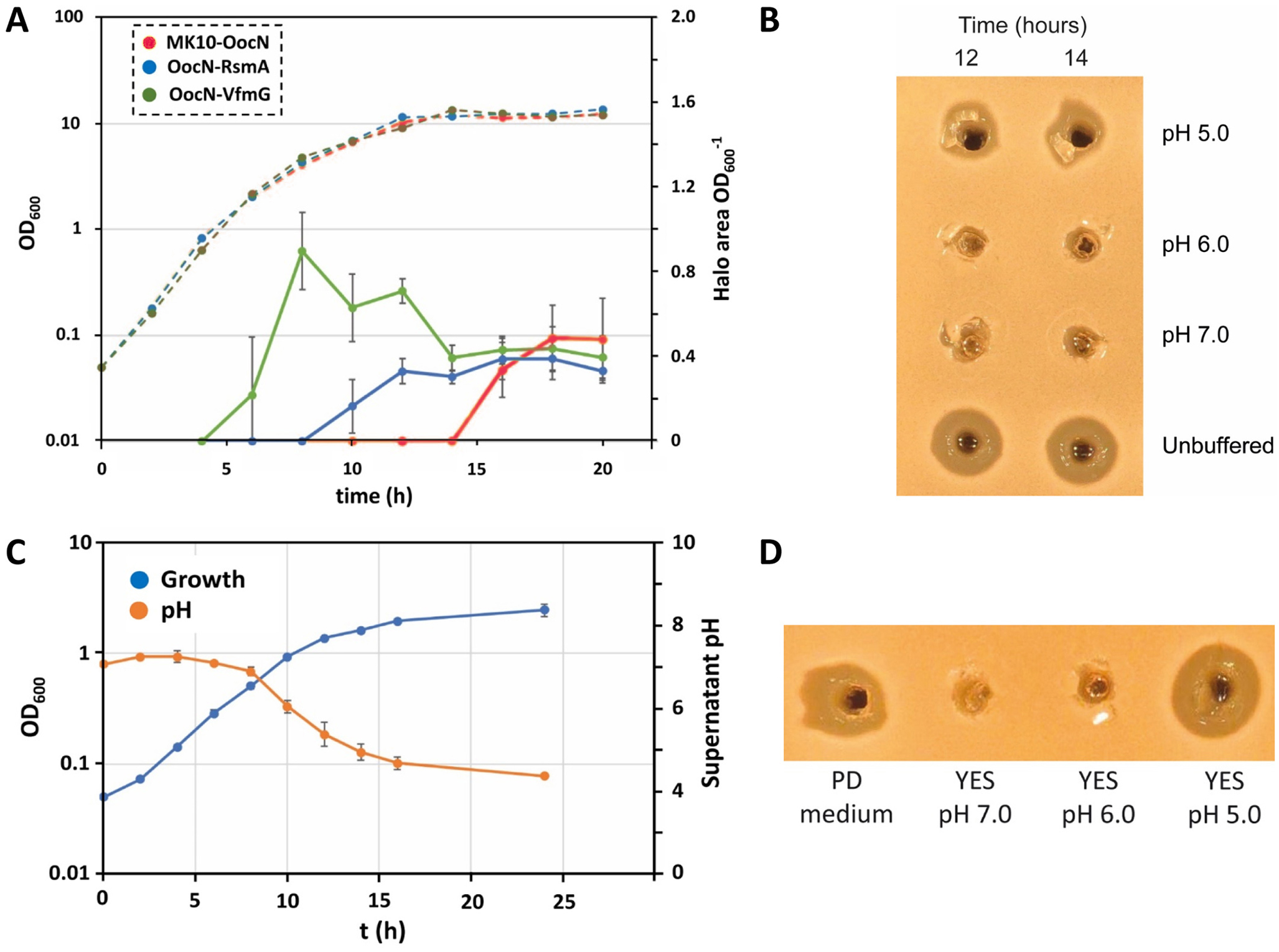

Supplement: FIG S7 [file mbio.02472-22-s0007.jpg]

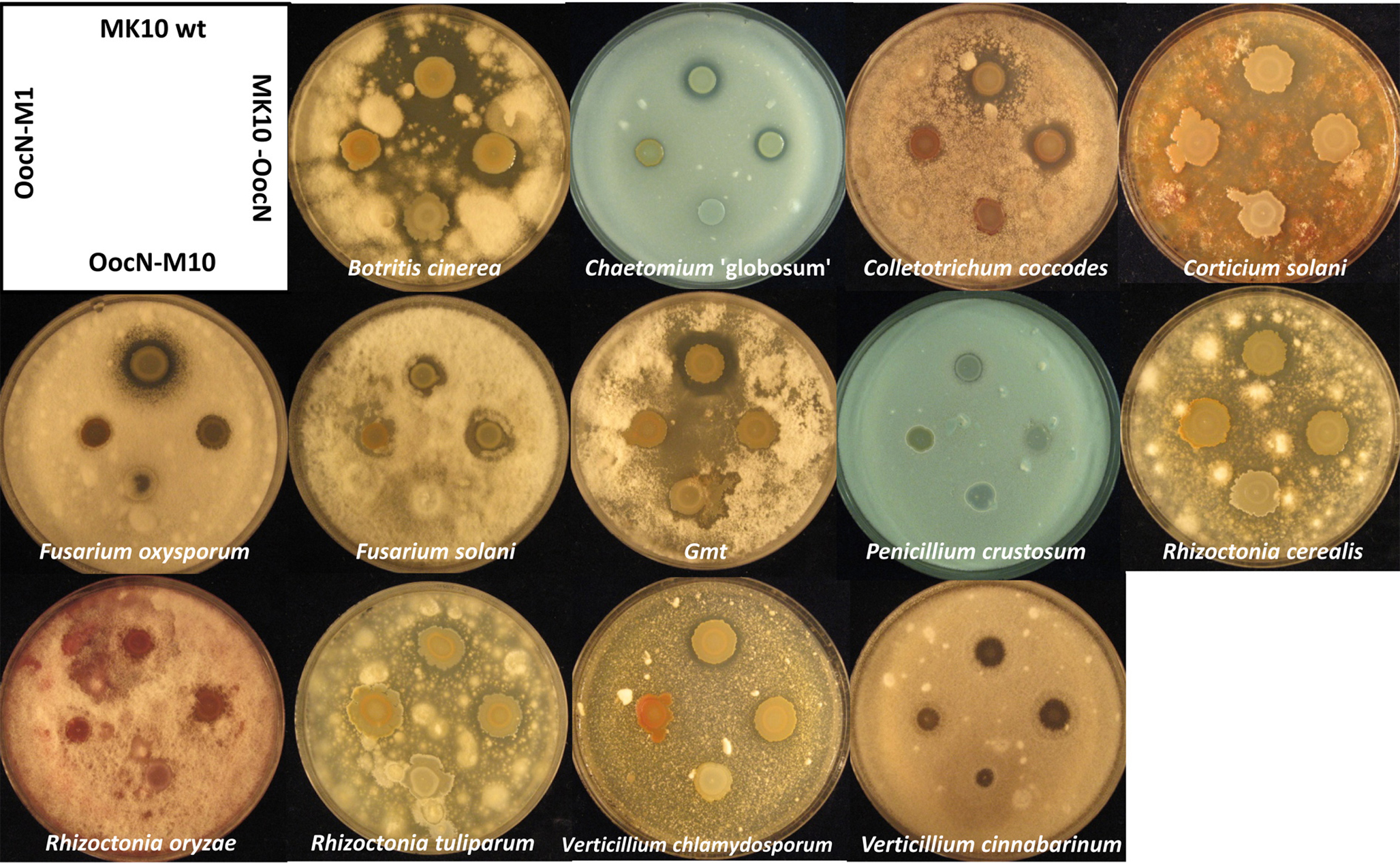

Supplement: FIG S8 [file mbio.02472-22-s0008.jpg]

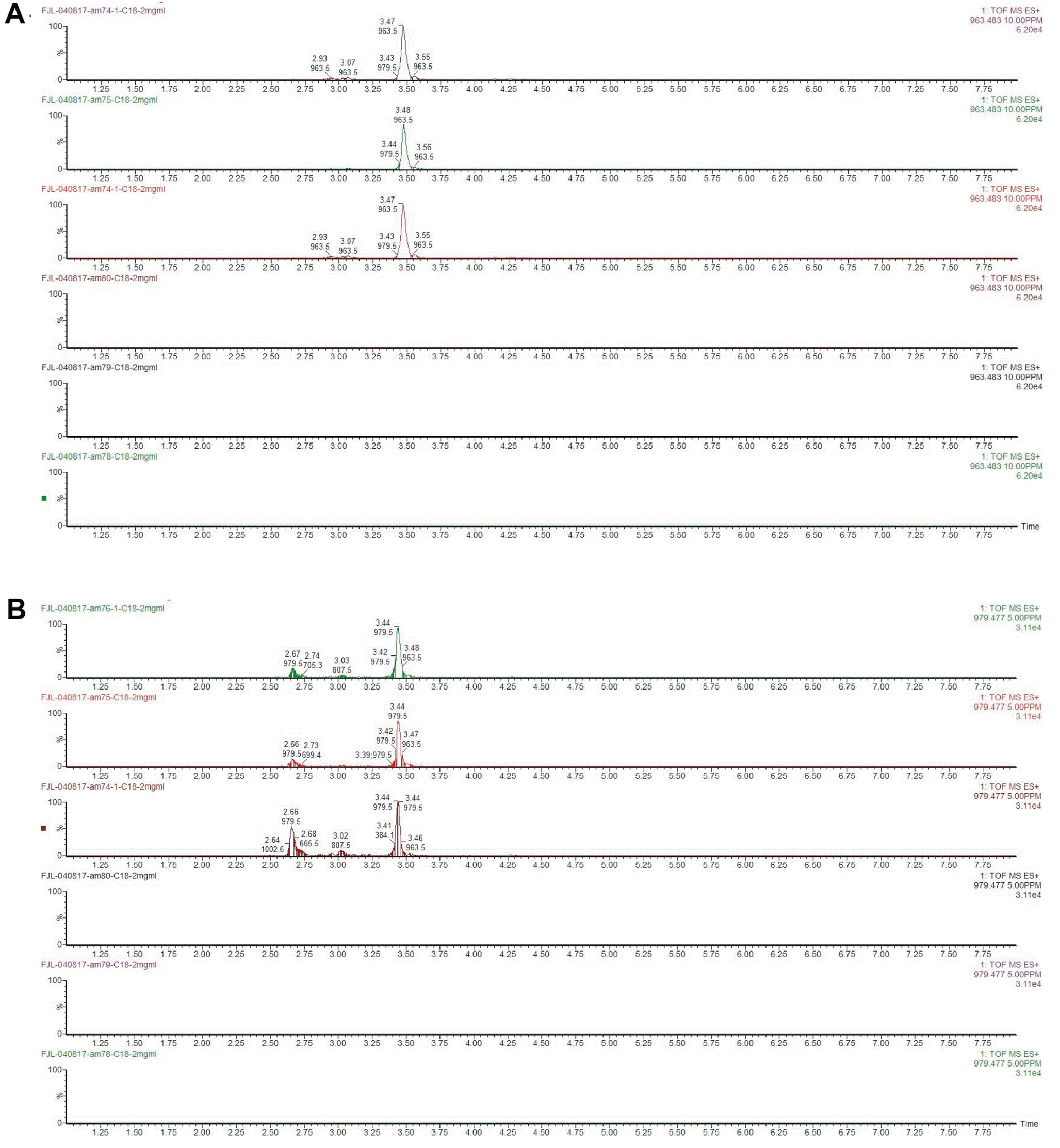

Supplement: FIG S9 [file mbio.02472-22-s0009.jpg]
