# Supplementary material for: Solanimycin: Biosynthesis and Distribution of a New Antifungal Antibiotic Regulated by Two Quorum-Sensing Systems
Source: mBio. 2022 Oct 10;13(6):e02472-22. doi: 10.1128/mbio.02472-22 (PMC9765074; doi:10.1128/mbio.02472-22)
Supplement: TABLE S1 [file mbio.02472-22-s0010.docx]

**Supplementary Table S1. Bacteria, oomycete, fungi, phages, plasmids and oligonucleotides used in this study**

| **Bacterial strains** | | **Genotype or relevant characteristic^a^** | | **Reference or source** | | |
| --- | --- | --- | --- | --- | --- | --- |
| *Escherichia coli* DH5α | | *supE44 lacU169*(*Ø80lacZΔ M15*) *hsdR17* (r_K_^−^m_K_^−^) *recA1* *endA1* *gyrA96 thi-1* *relA1* | | (1) | | |
| *E. coli* CC118λpir | | *araD*, Δ(*ara*, *leu*), Δ*lacZ*74, *pho*A20, *galK*, *thi-1*, *rspE*, *rpoB*, *argE*, *recA1*, λ*pir* | | (2) | | |
| *E. coli* HH26 | | Mobilizing strain for conjugal transfer | | (3) | | |
| *E. coli* β2163 | | F^-^ RP4-2-Tc::Mu ∆*dapA*::(*erm-pir*); Km^R^ Em^R^ | | (4) | | |
| *E. coli* OP50 | | Uracil auxotroph | | Lab stock | | |
| *Dickeya zeae* NCPPB3532 | | Wild type, plant pathogen | | James Hutton Institute | | |
| *Dickeya dadantii* 402 | | Wild type, plant pathogen | | James Hutton Institute | | |
| *Dickeya dadantii* 3937 | | Wild type, plant pathogen | | James Hutton Institute | | |
| *Dickeya dadantii* 703 | | Wild type, plant pathogen | | James Hutton Institute | | |
| *Dickeya dianthicola* 453 | | Wild type, plant pathogen | | James Hutton Institute | | |
| *Dickeya* *dianthicola* NCPPB3535 | | Wild type, plant pathogen | | James Hutton Institute | | |
| *Dickeya dianthicola* IPO980 | | Wild type, plant pathogen | | James Hutton Institute | | |
| *Dickeya paradisiaca* 2511 | | Wild type, plant pathogen | | James Hutton Institute | | |
| *Dickeya* sp. CSLRW240 | | Wild type, plant pathogen | | James Hutton Institute | | |
| *Dickeya* sp. NCPPB3274 | | Wild type, plant pathogen | | James Hutton Institute | | |
| *Dickeya* sp. MK7 | | Wild type, plant pathogen | | James Hutton Institute | | |
| *Dickeya solani* MK16 | | Wild type, plant pathogen | | (5) | | |
| *Dickeya solani* IPO_2222 | | Wild type, plant pathogen | | (5) | | |
| *Dickeya solani* MK10 | | Wild type, plant pathogen | | (5) | | |
| MK10-OocF | | MK10 transposon mutant *oocF*::Tn-KRCPN1; oocydin A negative, Km^R^ | | (6) | | |
| MK10-OocG | | MK10 transposon mutant *oocG*::Tn-KRCPN1; oocydin A negative, Km^R^ | | (6) | | |
| MK10-OocN | | MK10 transposon mutant *oocN*::Tn-KRCPN1; oocydin A negative, Km^R^ | | (6) | | |
| OocN-M1 | | MK10 transposon mutant *oocN*::Tn-KRCPN1, *solJ*::mini-Tn*5*Sm/Sp; Km^R^, Sm^R^ | | This study | | |
| OocN-M3 | | MK10 transposon mutant *oocN*::Tn-KRCPN1, *solA*::mini-Tn*5*Sm/Sp; Km^R^, Sm^R^ | | This study | | |
| OocN-M5 | | MK10 transposon mutant *oocN*::Tn-KRCPN1, *solA*::mini-Tn*5*Sm/Sp; Km^R^, Sm^R^ | | This study | | |
| OocN-M7 | | MK10 transposon mutant *oocN*::Tn-KRCPN1, *solH*::mini-Tn*5*Sm/Sp; Km^R^, Sm^R^ | | This study | | |
| OocN-M8 | | MK10 transposon mutant *oocN*::Tn-KRCPN1, *solK*::mini-Tn*5*Sm/Sp; Km^R^, Sm^R^ | | This study | | |
| OocN-M9A | | MK10 transposon mutant *oocN*::Tn-KRCPN1, *solA*::mini-Tn*5*Sm/Sp; Km^R^, Sm^R^ | | This study | | |
| OocN-M9B | | MK10 transposon mutant *oocN*::Tn-KRCPN1, intergenic region *solJ-solI*::mini-Tn*5*Sm/Sp; Km^R^, Sm^R^ | | This study | | |
| OocN-M10 | | MK10 transposon mutant *oocN*::Tn-KRCPN1, *solA*::mini-Tn*5*Sm/Sp; Km^R^, Sm^R^ | | This study | | |
| OocN-M11 | | MK10 transposon mutant *oocN*::Tn-KRCPN1, *solH*::mini-Tn*5*Sm/Sp; Km^R^, Sm^R^ | | This study | | |
| OocN-M12 | | MK10 transposon mutant *oocN*::Tn-KRCPN1, *solA*::mini-Tn*5*Sm/Sp; Km^R^, Sm^R^ | | This study | | |
| OocN-M14A | | MK10 transposon mutant *oocN*::Tn-KRCPN1, *solA*::mini-Tn*5*Sm/Sp; Km^R^, Sm^R^ | | This study | | |
| OocN-M14B | | MK10 transposon mutant *oocN*::Tn-KRCPN1, *solI*::mini-Tn*5*Sm/Sp; Km^R^, Sm^R^ | | This study | | |
| OocN-M16 | | MK10 transposon mutant *oocN*::Tn-KRCPN1, *solI*::mini-Tn*5*Sm/Sp; Km^R^, Sm^R^ | | This study | | |
| OocN-M17 | | MK10 transposon mutant *oocN*::Tn-KRCPN1, *solA*::mini-Tn*5*Sm/Sp; Km^R^, Sm^R^ | | This study | | |
| OocN-∆*solA* | | MK10 in-frame Δ*solA* (828 bp Δ), *oocN*::Tn-KRCPN1; Km^R^ | | This study | | |
| OocN-∆*solB* | | MK10 in-frame Δ*solB* (828 bp Δ), *oocN*::Tn-KRCPN1; Km^R^ | | This study | | |
| OocN-∆*solC* | | MK10 in-frame Δ*solC* (1038 bp Δ), *oocN*::Tn-KRCPN1; Km^R^ | | This study | | |
| OocN-∆*solD* | | MK10 in-frame Δ*solD* (210 bp Δ), *oocN*::Tn-KRCPN1; Km^R^ | | This study | | |
| OocN-∆*solE* | | MK10 in-frame Δ*solE* (1701 bp Δ), *oocN*::Tn-KRCPN1; Km^R^ | | This study | | |
| OocN-∆*solF* | | MK10 in-frame Δ*solF* (1701 bp Δ), *oocN*::Tn-KRCPN1; Km^R^ | | This study | | |
| OocN-∆*solG* | | MK10 in-frame Δ*solG* (1701 bp Δ), *oocN*::Tn-KRCPN1; Km^R^ | | This study | | |
| OocN-∆*solH* | | MK10 in-frame Δ*solH* (1701 bp Δ), *oocN*::Tn-KRCPN1; Km^R^ | | This study | | |
| OocN-∆*solI* | | MK10 in-frame Δ*solI* (1086 bp Δ), *oocN*::Tn-KRCPN1; Km^R^ | | This study | | |
| OocN-∆*solJ* | | MK10 in-frame Δ*solJ* (492 bp Δ), *oocN*::Tn-KRCPN1; Km^R^ | | This study | | |
| OocN-∆*solK* | | MK10 in-frame Δ*solK* (387 bp Δ), *oocN*::Tn-KRCPN1; Km^R^ | | This study | | |
| OocN-∆*solL* | | MK10 in-frame Δ*solL* (1170 bp Δ), *oocN*::Tn-KRCPN1; Km^R^ | | This study | | |
| MK10Lac | | *D. solani* MK10 *lacZ*::mini-Tn*5*Sm/Sp, Sm^R^ | | This study | | |
| MK10Lac-SolA | | *D. solani* MK10 *lacZ*::mini-Tn*5* Sm/Sp, *solA*::Tn-KRCPN1; Sm^R^, Km^R^. *solA* transcriptional fusion. | | This study | | |
| MK10-RsmA | | *D. solani* MK10 *rsmA*::Tn-DS1028; Cm^R^ | | This study | | |
| OocN-RsmA | | *D. solani* MK10 *lacZ*::mini-Tn*5*, *rsmA*::Tn-DS1028; *oocN*::Tn-KRCPN1; Km^R^, Sm^R^, Cm^R^ | | This study | | |
| REM666 | | *D. solani* MK10 *lacZ*::mini-Tn5, *solA*::Tn-KRCPN1, *rsmA*::Tn-DS1028 Sm^R^, Km^R^, Cm^R^*. solA* transcriptional fusion. | | This study | | |
| MK10-ExpI | | *D. solani* MK10 *expI*::Tn-DS1028; Cm^R^ | | This study | | |
| OocN-ExpI | | *D. solani* MK10 *lacZ*::mini-Tn*5*, *expI*::Tn-DS1028; *oocN*::Tn-KRCPN1; Km^R^, Sm^R^, Cm^R^ | | This study | | |
| MK10-ExpI-SolA | | *D. solani* MK10 *expI*::Tn-DS1028 , *lacZ*::mini-Tn5 Sm/Sp, *solA*::Tn-KRCPN1; Sm^R^, Km^R^, Cm^R^*. solA* transcriptional fusion. | | This study | | |
| MK10-VfmG | | *D. solani* MK10 *vfmG*::Tn-DS1028 Cm^R^. | | This study | | |
| OocN-VfmG | | *D. solani* MK10 *lacZ*::mini-Tn*5*, *vfmG*::Tn-DS1028; *oocN*::Tn-KRCPN1; Km^R^, Sm^R^, Cm^R^ | | This study | | |
| MK10-VfmG-SolA | | *D. solani* MK10 *vfmG*::Tn-DS1028 , *lacZ*::mini-Tn5 Sm/Sp, *solA*::Tn-KRCPN1; Sm^R^, Km^R^, Cm^R^*. solA* transcriptional fusion. | | This study | | |
| MK10-M10 | | MK10 transposon mutant *solA*::mini-Tn*5*Sm/Sp; Sm^R^ | | This study | | |
| MK16-OocN | | MK16 transposon mutant *oocN*::Tn-KRCPN1; oocydin A negative, Km^R^ | | This study | | |
| 16OocN-M10 | | MK16 transposon mutant *oocN*::Tn-KRCPN1, *solA*::mini-Tn*5*Sm/Sp; Km^R^, Sm^R^ | | This study | | |
| 16OocN-M11 | | MK16 transposon mutant *oocN*::Tn-KRCPN1, *solH*::mini-Tn*5*Sm/Sp; Km^R^, Sm^R^ | | This study | | |
| 16OocN-M14A | | MK16 transposon mutant *oocN*::Tn-KRCPN1, *solA*::mini-Tn*5*Sm/Sp; Km^R^, Sm^R^ | | This study | | |
| 16OocN-M16 | | MK16 transposon mutant *oocN*::Tn-KRCPN1, *solI*::mini-Tn*5*Sm/Sp; Km^R^, Sm^R^ | | This study | | |
| IPO_2222-OocN | | IPO 2222 transposon mutant *oocN*::Tn-KRCPN1; oocydin A negative, Km^R^ | | This study | | |
| **Fungi/oomycete strains** | | **Genotype or relevant characteristic^a^** | | **Reference or source** | | |
| *Saccharomyces cerevisiae* | | Wild type | | S. Oliver | | |
| *Schizosaccharomyces pombe* | | Wild type | | J. Mata | | |
| *Candida albicans* | | Wild type | | Lab stock | | |
| *Pythium ultimum* | | Wild type, plant pathogen | | C.A. Gilligan | | |
| *Verticillium dahliae* 5368 | | Wild type, plant pathogen | | R. Cooper | | |
| *Helminthosporium sativum* | | Wild type, plant pathogen | | C.A. Gilligan | | |
| *Pyrenophora graminae* | | Wild type, plant pathogen | | C.A. Gilligan | | |
| *Cladosporium* sp. *C45* | | Wild type, plant pathogen | | C.A. Gilligan | | |
| *Mycosphaerella graminicola* | | Wild type, plant pathogen | | C.A. Gilligan | | |
| *Penicillium crustosum* | | Wild type, plant pathogen | | C.A. Gilligan | | |
| *Phialophora fastigiata* | | Wild type, plant pathogen | | C.A. Gilligan | | |
| *Gaeumannomyces graminis* var. *tritici* (*Gmt*) | | Wild type, plant pathogen | | C.A. Gilligan | | |
| *Colletotrichum coccodes* | | Wild type, plant pathogen | | C.A. Gilligan | | |
| *Fusarium culmorum* | | Wild type, plant pathogen | | C.A. Gilligan | | |
| *Fusarium oxysporum* | | Wild type, plant pathogen | | C.A. Gilligan | | |
| *Fusarium solani* | | Wild type, plant pathogen | | C.A. Gilligan | | |
| *Chaetomium globosum* | | Wild type, plant pathogen | | C.A. Gilligan | | |
| *Verticillium cinnabarinum* | | Wild type, plant pathogen | | C.A. Gilligan | | |
| *Verticillium chlamydosporum* | | Wild type, plant pathogen | | C.A. Gilligan | | |
| *Botrytis allii* | | Wild type, plant pathogen | | C.A. Gilligan | | |
| *Botritis cinerea* | | Wild type, plant pathogen | | C.A. Gilligan | | |
| *Botrytis fabae* | | Wild type, plant pathogen | | C.A. Gilligan | | |
| *Monilinia fructigena* | | Wild type, plant pathogen | | C.A. Gilligan | | |
| *Armillaria mellea* | | Wild type, plant pathogen | | C.A. Gilligan | | |
| *Corticium solani* | | Wild type, plant pathogen | | C.A. Gilligan | | |
| *Rhizoctonia solani* | | Wild type, plant pathogen | | C.A. Gilligan | | |
| *Rhizoctonia cerealis* | | Wild type, plant pathogen | | C.A. Gilligan | | |
| *Rhizoctonia tuliparum* | | Wild type, plant pathogen | | C.A. Gilligan | | |
| *Rhizoctonia oryzae* | | Wild type, plant pathogen | | C.A. Gilligan | | |
| **Phages** | | | | | | |
| ϕXF1 | | Generalized transducing phage for *D. solani* strains MK10, MK16 and IPO 2222 | | (6) | | |
| **Plasmids** | | **Relevant characteristic^a^** | | **Source** | | |
| pKNG101 | | Sm^R^; *oriR6K mob sacBR* | | (3) | | |
| pUC18Not | | Ap^R^; identical to pUC18 but with two NotI sites flanking pUC18 polylinker | | (2) | | |
| pNJ5000 | | Tc^R;^ Mobilizing plasmid used in marker exchange | | (7) | | |
| pQE-80-L | | Ap^R;^ IPTG-inducible promoter, ColE1 origin. | | Qiagen | | |
| pTA100 | | Sm^R^, Sp^R^; pQE-80L-based expression vector with Ap^R^ resistance cassette replaced by Sm/Sp. IPTG-inducible promoter, ColE1 origin | | (8) | | |
| pBluescript II SK(+) | | Ap^R^; Cloning vector, ColE1 replicon | | Addgene | | |
| pUTmini-Tn*5-*Sm/Sp | | Ap^R^, Sm^R^, Sp^R^; Delivery plasmid for mini-Tn*5*Sm/Sp; Plasmid used in transposon mutagenesis | | (2) | | |
| pKRCPN1 | | Tc^R^, Km^R^; *tetA, tnp, ‘lacZ, oriR6K, aph;* Delivery plasmid for mini-Tn*5*Sm/Sp; Plasmid used in transposon mutagenesis. Used to generate the transcriptional fusion *solA*::*lacZ* | | (9) | | |
| pMAMV204 | | Ap^R^; 1.3-kb PCR product containing a 1170 bp in frame deletion of *solL* of MK10 inserted into the EcoRI/PstI sites of pUC18Not | | This study | | |
| pMAMV205 | | Ap^R^; 1.4-kb PCR product containing a 387 bp in frame deletion of *solK* of MK10 inserted into the EcoRI/PstI sites of pUC18Not | | This study | | |
| pMAMV206 | | Ap^R^; 1.4-kb PCR product containing a 492 bp in frame deletion of *solJ* of MK10 inserted into the EcoRI/PstI sites of pUC18Not | | This study | | |
| pMAMV207 | | Ap^R^; 1.4-kb PCR product containing a 1086 bp in frame deletion of *solI* of MK10 inserted into the EcoRI/PstI sites of pUC18Not | | This study | | |
| pMAMV208 | | Ap^R^; 1.4-kb PCR product containing a 1701 bp in frame deletion of *solE* of MK10 inserted into the EcoRI/PstI sites of pUC18Not | | This study | | |
| pMAMV209 | | Ap^R^; 1.5-kb PCR product containing a 210 bp in frame deletion of *solD* of MK10 inserted into the EcoRI/PstI sites of pUC18Not | | This study | | |
| pMAMV210 | | Ap^R^; 1.4-kb PCR product containing a 1038 bp in frame deletion of *solC* of MK10 inserted into the EcoRI/PstI sites of pUC18Not | | This study | | |
| pMAMV211 | | Ap^R^; 1.5-kb PCR product containing a 832 bp in frame deletion of *solB* of MK10 inserted into the EcoRI/PstI sites of pUC18Not | | This study | | |
| pMAMV212 | | Sm^R^; 1.4-kb NotI fragment of pMAMV204 was cloned at the same site in pKNG101 | | This study | | |
| pMAMV213 | | Sm^R^; 1.5-kb NotI fragment of pMAMV205 was cloned at the same site in pKNG101 | | This study | | |
| pMAMV214 | | Sm^R^; 1.5-kb NotI fragment of pMAMV206 was cloned at the same site in pKNG101 | | This study | | |
| pMAMV215 | | Sm^R^; 1.5-kb NotI fragment of pMAMV207 was cloned at the same site in pKNG101 | | This study | | |
| pMAMV216 | | Sm^R^; 1.5-kb NotI fragment of pMAMV208 was cloned at the same site in pKNG101 | | This study | | |
| pMAMV217 | | Sm^R^; 1.6-kb NotI fragment of pMAMV209 was cloned at the same site in pKNG101 | | This study | | |
| pMAMV218 | | Sm^R^; 1.5-kb NotI fragment of pMAMV210 was cloned at the same site in pKNG101 | | This study | | |
| pMAMV219 | | Sm^R^; 1.6-kb NotI fragment of pMAMV211 was cloned at the same site in pKNG101 | | This study | | |
| pSolB | | Sm^R^; *solB* gene was cloned into the BamHI/HindIII sites of pTA100 | | This study | | |
| pREM281 | | Ap^R^; 1.0-kb PCR product containing an in-frame deletion of *solA* of MK10 inserted into the ApaI/SpeI sites of pBluescript | | This study | | |
| pREM282 | | Ap^R^; 1.1-kb PCR product containing an in-frame deletion of *solF* of MK10 inserted into the ApaI/SpeI sites of pBluescript | | This study | | |
| pREM283 | | Ap^R^; 1.0-kb PCR product containing an in-frame deletion of *solG* of MK10 inserted into the ApaI/SpeI sites of pBluescript | | This study | | |
| pREM284 | | Ap^R^; 1.0-kb PCR product containing an in-frame deletion of *solH* of MK10 inserted into the ApaI/SpeI sites of pBluescript | | This study | | |
| pREM288 | | Sm^R^; 1.0-kb ApaI/SpeI fragment of pREM281 was cloned at the same site in pKNG101 | | This study | | |
| pREM289 | | Sm^R^; 1.0-kb ApaI/SpeI fragment of pREM282 was cloned at the same site in pKNG101 | | This study | | |
| pREM290 | | Sm^R^; 1.0-kb ApaI/SpeI fragment of pREM283 was cloned at the same site in pKNG101 | | This study | | |
| pREM291 | | Sm^R^; 1.0-kb ApaI/SpeI fragment of pREM284 was cloned at the same site in pKNG101 | | This study | | |
| pSolC | | Sm^R^; *solC* gene was cloned into the BamHI/HindIII sites of pTA100 | | This study | | |
| pSolD | | Sm^R^; *solD*  gene was cloned into the BamHI/HindIII sites of pTA100 | | This study | | |
| pSolI | | Sm^R^, *solI* gene was cloned into the BamHI/HindIII sites of pTA100 | | This study | | |
| pSolK | | Sm^R^; *solK* gene was cloned into the BamHI/HindIII sites of pTA100 | | This study | | |
| pQE80-ExpI | | Ap^R^; *expI* gene was cloned into the EcoRI/PstI sites of pQE80-L | | This study | | |
| **Oligonucleótides** | | | | | |  |
| **Name** | **Sequence (5´- 3')** | | **Description** | | **Source** |  |
| MAMV3 | CTAAGCTGATCCGGTGGATG | | mini-Tn*5*Sm/Sp specific primer | | (6) |  |
| MAMV4 | AACGGTTTACAAGCATAAAGC | | mini-Tn*5*Sm/Sp specific primer | | (6) |  |
| PF106 | GACCACACGTCGACTAGTGCNNNNNNNNNNAGAG | | Random primed PCR primer 1 | | (10) |  |
| PF107 | GACCACACGTCGACTAGTGCNNNNNNNNNNACGCC | | Random primed PCR primer 2 | | (10) |  |
| PF108 | GACCACACGTCGACTAGTGCNNNNNNNNNNGATAC | | Random primed PCR primer 3 | | (10) |  |
| PF109 | GACCACACGTCGACTAGTGC | | Random primed PCR adapter primer | | (10) |  |
| SolL-EcoRI-F | TAATGAATTCAGCACATAGTGCCCAGAGC | | Forward primer to clone downstream flanking region of *solL* for in-frame deletion | | This study |  |
| SolL-BamHI-R | TAATGGATCCGAAAGCTCCAGGTTGCGTTC | | Reverse primer to clone downstream flanking region of *solL* for in-frame deletion | | This study |  |
| SolL-BamHI-F | TAATGGATCCGCTTACCGATATTGCCGCC | | Forward primer to clone upstream flanking region of *solL* for in-frame deletion | | This study |  |
| SolL-PstI-R | TAATCTGCAGAAGCTTACCTCTGGCGCAC | | Reverse primer to clone upstream flanking region of *solL* for in-frame deletion | | This study |  |
| SolK-EcoRI-F | TAATGAATTCCCAGACCACTAATCAGAACGC | | Forward primer to clone downstream flanking region of *solK* for in-frame deletion | | This study |  |
| SolK-BamHI-R | TAATGGATCCCGTCAAGAGTTACTCGGTAAGC | | Reverse primer to clone downstream flanking region of *solK* for in-frame deletion | | This study |  |
| SolK-BamHI-F | TAATGGATCCCAGCTTTGGTGGTGTCACATC | | Forward primer to clone upstream flanking region of *solK* for in-frame deletion | | This study |  |
| SolK-PstI-R | TAATCTGCAGACCCATGCTCATCCTGACC | | Reverse primer to clone upstream flanking region of *solK* for in-frame deletion | | This study |  |
| SolJ-EcoRI-F | TAATGAATTCTCCGGCTTGGAAGAGGATACCT | | Forward primer to clone downstream flanking region of *solJ* for in-frame deletion | | This study |  |
| SolJ-BamHI-R | TAATGGATCCGTCCGCATTTATCCTGGGC | | Reverse primer to clone downstream flanking region of *solJ* for in-frame deletion | | This study |  |
| SolJ-BamHI-F | TAATGGATCCCGTCACCGCAATATCGTCTG | | Forward primer to clone upstream flanking region of *solJ* for in-frame deletion | | This study |  |
| SolJ-PstI-R | TAATCTGCAGCACGATGTTGAAATCCGTGGC | | Reverse primer to clone upstream flanking region of *solJ* for in-frame deletion | | This study |  |
| SolI-EcoRI-F | TAATGAATTCGCCTCCTTACGCGATATCCAC | | Forward primer to clone downstream flanking region of *solI* for in-frame deletion | | This study |  |
| SolI-BamHI-R | TAATGGATCCGTACTTGAAGAGCCGGAGCG | | Reverse primer to clone downstream flanking region of *solI* for in-frame deletion | | This study |  |
| SolI-BamHI-F | TAATGGATCCCTCGAACATCGGCAATTCAGC | | Forward primer to clone upstream flanking region of *solI* for in-frame deletion | | This study |  |
| SolI-PstI-R | TAATCTGCAGCGCTGATCGATTCAGAAGCG | | Reverse primer to clone upstream flanking region of *solI* for in-frame deletion | | This study |  |
| SolE-EcoRI-F | TAATGAATTCCATCTGCCTCGGTCATCTCG | | Forward primer to clone downstream flanking region of *solE* for in-frame deletion | | This study |  |
| SolE-BamHI-R | TAATGGATCCAGGAACGCCGAAAGCGTATC | | Reverse primer to clone downstream flanking region of *solE* for in-frame deletion | | This study |  |
| SolE-BamHI-F | TAATGGATCCGATCTCATCGGCCTCGCTG | | Forward primer to clone upstream flanking region of *solE* for in-frame deletion | | This study |  |
| SolE-PstI-R | TAATCTGCAGCATTCTTCGCGGAGAGTCCG | | Reverse primer to clone upstream flanking region of *solE* for in-frame deletion | | This study |  |
| SolD-EcoRI-F | TAATGAATTCCGCATTGAGCGGATTGAAAGT | | Forward primer to clone downstream flanking region of *solD* for in-frame deletion | | This study |  |
| SolD-BamHI-R | TAATGGATCCGAAGCCCATCTACCGGCTG | | Reverse primer to clone downstream flanking region of *solD* for in-frame deletion | | This study |  |
| SolD-BamHI-F | TAATGGATCCGAGAATGTCTTTCTGAGCGTCCA | | Forward primer to clone upstream flanking region of *solD* for in-frame deletion | | This study |  |
| SolD-HindIII-R | TAATAAGCTTGCGCGTTCGAAACCTCATAC | | Reverse primer to clone upstream flanking region of *solD* for in-frame deletion | | This study |  |
| SolC-EcoRI-F | TAATGAATTCGCGCATCCAATCCGCTTTG | | Forward primer to clone downstream flanking region of *solC* for in-frame deletion | | This study |  |
| SolC-BamHI-R | TAATGGATCCCGTCTCTCCGTGATGTCTTCC | | Reverse primer to clone downstream flanking region of *solC* for in-frame deletion | | This study |  |
| SolC-BamHI-F | TAATGGATCCCTGGCGTCCGAACATGAAG | | Forward primer to clone upstream flanking region of *solC* for in-frame deletion | | This study |  |
| SolC-HindIII-R | TAATAAGCTTCGCTGAGGAGCTGGTGTAC | | Reverse primer to clone upstream flanking region of *solC* for in-frame deletion | | This study |  |
| SolB-EcoRI-F | TAATGAATTCGAATGGTGATGATGTCGTGCG | | Forward primer to clone downstream flanking region of *solB* for in-frame deletion | | This study |  |
| SolB-BamHI-R | TAATGGATCCCAGATGGTGGAGGAGTGCG | | Reverse primer to clone downstream flanking region of *solB* for in-frame deletion | | This study |  |
| SolB-BamHI-F | TAATGGATCCGGATGACGCTCGCACATACG | | Forward primer to clone upstream flanking region of *solB* for in-frame deletion | | This study |  |
| SolB-HindIII-R | TAATAAGCTTCTGTACCCAACGTCAGGCG | | Reverse primer to clone upstream flanking region of *solB* for in-frame deletion | | This study |  |
| oREM668 | Ccgggcccgaataaataagattcttgtgc  GCGATCCCTT CGCAATAACTGATGTCATTTG  ctctggcgtaatcgccgtcgtctc  GG ACTAGT GTCGGGTTCAGAATGACGG | | Forward primer to clone downstream flanking region of *solA* for in-frame deletion | | This study |  |
| oREM669 | GCGATCCCTT CGCAATAACTGATGTCATTTG | | Reverse primer to clone downstream flanking region of *solA* for in-frame deletion | | This study |  |
| oREM670 | ctctggcgtaatcgccgtcgtctc | | Forward primer to clone upstream flanking region of *solA* for in-frame deletion | | This study |  |
| oREM671 | GGACTAGTGTCGGGTTCAGAATGACGG | | Reverse primer to clone upstream flanking region of *solA* for in-frame deletion | | This study |  |
| oREM694  oREM695  oREM696  oREM697 | Ccgggccccgcacttccacgcgaatgccg | | Forward primer to clone downstream flanking region of *solF* for in-frame deletion | | This study |  |
| oREM695 | GGGTCGTTTCCTGCATGGTAGCCATTGG | | Reverse primer to clone downstream flanking region of *solF* for in-frame deletion | | This study |  |
| oREM696 | catgcaggaaacgaccctggccgtttcgttg | | Forward primer to clone upstream flanking region of *solF* for in-frame deletion | | This study |  |
| oREM697 | GGACTAGTGTCGATACGCCTTCCCAGTAG | | Reverse primer to clone upstream flanking region of *solF* for in-frame deletion | | This study |  |
| oREM698 | Ccgggcccaggtcgcctgtttgatcgattg | | Forward primer to clone downstream flanking region of *solG* for in-frame deletion | | This study |  |
| oREM699  oREM695  oREM696  oREM697 | GGGCTATCGAATATTCGATGGTGATTGACG | | Reverse primer to clone downstream flanking region of *solG* for in-frame deletion | | This study |  |
| oREM700 | ccatcgaatattcgatagcccgacgttagcc | | Forward primer to clone upstream flanking region of *solG* for in-frame deletion | | This study |  |
| oREM701 | GGACTAGTGCCGAGCGCGACAAACCGCGCG | | Reverse primer to clone upstream flanking region of *solG* for in-frame deletion | | This study |  |
| oREM702  o | Ccgggccctcgtatcgaattgggcgagatcg | | Forward primer to clone downstream flanking region of *solH* for in-frame deletion | | This study |  |
| oREM703 | GCAAACCTTTATTCGATCATAATCTCATCTCATCG | | Reverse primer to clone downstream flanking region of *solH* for in-frame deletion | | This study |  |
| oREM704 | atgatcgaataaaggtttgcgtctgcatacc | | Forward primer to clone upstream flanking region of *solH* for in-frame deletion | | This study |  |
| oREM705 | GGACTAGTGCTCACGCATAATCGGCTCCA | | Reverse primer to clone upstream flanking region of *solH* for in-frame deletion | | This study |  |
| oREM672  oREM673  oREM674  oREM675  oREM676  oREM677  oREM678  oREM679  oREM680  oREM681  oREM682  oREM683  oREM684  oREM685  oREM686  oREM687  oREM688  oREM689  oREM690  oREM691  oREM692  oREM693 | cgttgagacgctgatggccgctggct  gcgacaccccgcatgccagcgccgcc  gcgaatgctcgctataccgagct  cagcagcaccgcgtcttcgtg | | RT-PCR oligo forward *solA-solB* | | This study |  |
| oREM673  o | gcgacaccccgcatgccagcgccgcc | | RT-PCR oligo reverse *solA-solB* | | This study |  |
| oREM674 | gcgaatgctcgctataccgagct | | RT-PCR oligo forward *solB-solC* | | This study |  |
| oREM675 | cagcagcaccgcgtcttcgtg | | RT-PCR oligo reverse *solB-solC* | | This study |  |
| oREM676 | tcttcgcggagagtccgggatc | | RT-PCR oligo forward *solC-solD* | | This study |  |
| oREM677 | ccagatccccgagcgtgtaaatac | | RT-PCR oligo reverse *solC-solD* | | This study |  |
| oREM678 | cgtggacctggattcatttatcgg | | RT-PCR oligo forward *solD-solE* | | This study |  |
| oREM679 | cgtctcgcaggcaacgatgtcatcat | | RT-PCR oligo reverse *solD-solE* | | This study |  |
| oREM680 | gctttatcagggcgccgagttagc | | RT-PCR oligo forward *solE-solF* | | This study |  |
| oREM681 | ccactcatgcataacgcgttcg | | RT-PCR oligo reverse *solE-solF* | | This study |  |
| oREM682 | gccggtgttaatgaggtcgcctgt | | RT-PCR oligo forward *solF-solG* | | This study |  |
| oREM683 | ctctggcgtaatcgccgtcgtctc | | RT-PCR oligo reverse *solF-solG* | | This study |  |
| oREM684 | gattatctcgggcgtaacgataacc | | RT-PCR oligo forward *solG-solH* | | This study |  |
| oREM685 | cttgtcgctcaactggtgcaga | | RT-PCR oligo reverse *solG-solH* | | This study |  |
| oREM686 | cgatgcccgttcacctgcttggc | | RT-PCR oligo forward *solH-solI* | | This study |  |
| oREM687 | gatagttatttccaacaacgcg | | RT-PCR oligo reverse *solH-solI* | | This study |  |
| oREM688 | ccgataccttcagcatcgaccgtg | | RT-PCR oligo forward *solI-solJ* | | This study |  |
| oREM689 | cgatgtttatttgccaaatactcag | | RT-PCR oligo reverse *solI-solJ* | | This study |  |
| oREM690 | ggtttaacgtttgaacaagta | | RT-PCR oligo forward *solJ-solK* | | This study |  |
| oREM691 | gattcatggtaatacttactg | | RT-PCR oligo reverse *solJ-solK* | | This study |  |
| oREM692 | caactggcttaggcctacccgac | | RT-PCR oligo forward *solK-solL* | | This study |  |
| oREM693 | ccggctatactcaagccaatgcc | | RT-PCR oligo reverse *solK-solL* | | This study |  |
| C-SolB-BamHI-F | TAATGGATCCAAAATACTGATCACCGGAGCA | | Forward primer to clone *solB* into pTA100 | | This study |  |
| C-SolB-HindIII-R | TAATAAGCTTGAGAGAGATTCCGATTGCTAAGG | | Reverse primer to clone *solB* into pTA100 | | This study |  |
| C-SolC-BamHI-F | TAATGGATCCGATTTTCATGATCTGATCGAACTTG | | Forward primer to clone *solC* into pTA100 | | This study |  |
| C-SolC-HindIII-R | TAATAAGCTTCGAGAACAACGGAGAGAATG | | Reverse primer to clone *solC* into pTA100 | | This study |  |
| C-SolD-BamHI-F | TAATGGATCCGACGCTCAGAAAGACATTCTC | | Forward primer to clone *solD* into pTA100 | | This study |  |
| C-SolD-HindIII-R | TAATAAGCTTGCTTGTTTATCCCAACAGCG | | Reverse primer to clone *solD* into pTA100 | | This study |  |
| C-SolE-BamHI-F | TAATGGATCCTCTTCTTCAGTTTGGGTATTTCC | | Forward primer to clone *solE* into pTA100 | | This study |  |
| C-OrfE-HindIII-R | TAATAAGCTTAGTATCCTTACGCGACAAGAG | | Reverse primer to clone *solE* into pTA100 | | This study |  |
| C-SolI-BamHI-F | GCGGGATCCATGACTAAACAATTACAGGATG | | Forward primer to clone *solI* into pTA100 | | This study |  |
| C-SolI-HindIII-R | CCCAAGCTTTCACGCTTTAGGAATGATGAC | | Reverse primer to clone *solI* into pTA100 | | This study |  |
| C-SolK-BamHI-F | TAATGGATCCCATGATGGAAATTATTCAAACGACT | | Forward primer to clone *solK* into pTA100 | | This study |  |
| C-SolK-SphI-R | TAATGCATGCAATGCATGACCTTCCCATCC | | Reverse primer to clone *solK* into pTA100 | | This study |  |
| C-ExpI-EcoRI-F | CGCGGAATTCATGACAGGGGGAAATATGTT | | Forward primer to clone *expI* into pQE-80-L | | This study |  |
| C-ExpI-PstI-R | CGCGCTGCAGAATCTGATCAAGCCAGTGGGA | | Forward primer to clone *expI* into pQE-80-L | | This study |  |

### *^a^*Ap, ampicillin; Cm, [Chloramphenicol](http://en.wikipedia.org/wiki/Chloramphenicol); Em, erythromycin; Km, kanamycin; Sm, streptomycin; Sp, spectinomycin; Tc, tetracycline.

**REFERENCES**

1. Woodcock DM, Crowther PJ, Doherty J, Jefferson S, DeCruz E, Noyer-Weidner M, Smith SS, Michael MZ, Graham MW. 1989. Quantitative evaluation of *Escherichia coli* host strains for tolerance to cytosine methylation in plasmid and phage recombinants. Nucleic Acids Res 17:3469–3478.

2. Herrero M, de Lorenzo V, Timmis KN. 1990. Transposon vectors containing non-antibiotic resistance selection markers for cloning and stable chromosomal insertion of foreign genes in gram-negative bacteria. J Bacteriol 172:6557–6567.

3. Kaniga K, Delor I, Cornelis GR. 1991. A wide-host-range suicide vector for improving reverse genetics in gram-negative bacteria: inactivation of the *blaA* gene of *Yersinia enterocolitica.* Gene 109:137–141.

4. Demarre G, Guerout AM, Matsumoto-Mashimo C, Rowe-Magnus DA, Marliere P, Mazel D. 2005. A new family of mobilizable suicide plasmids based on broad host range R388 plasmid (IncW) and RP4 plasmid (IncPalpha) conjugative machineries and their cognate *Escherichia coli* host strains. Res Microbiol 156:245–255.

5. Pritchard L, Humphris S, Baeyen S, Maes M, Van Vaerenbergh J, Elphinstone J, Saddler G, Toth I. 2013. Draft Genome Sequences of Four *Dickeya dianthicola* and Four *Dickeya solani* Strains. Genome Announc 1:e00087-12.

6. Matilla MA, Fang X, Salmond GP. 2014. Viunalikeviruses are environmentally common agents of horizontal gene transfer in pathogens and biocontrol bacteria. ISME J 8:2143–2147.

7. Grinter NJ. 1983. A broad-host-range cloning vector transposable to various replicons. Gene 21:133–143.

8. Fineran PC, Blower TR, Foulds IJ, Humphreys DP, Lilley KS, Salmond GP. 2009. The phage abortive infection system, ToxIN, functions as a protein-RNA toxin-antitoxin pair. Proc Natl Acad Sci U S A 106:894–899.

9. Monson R, Smith DS, Matilla MA, Roberts K, Richardson E, Drew A, Williamson N, Ramsay J, Welch M, Salmond GP. 2015. A Plasmid-Transposon Hybrid Mutagenesis System Effective in a Broad Range of Enterobacteria. Front Microbiol 6:1442.

10. Fineran PC, Everson L, Slater H, Salmond GP. 2005. A GntR family transcriptional regulator (PigT) controls gluconate-mediated repression and defines a new, independent pathway for regulation of the tripyrrole antibiotic, prodigiosin, in *Serratia*. Microbiology 151:3833–3845.
