# Supplementary material for: Solanimycin: Biosynthesis and Distribution of a New Antifungal Antibiotic Regulated by Two Quorum-Sensing Systems
Source: mBio. 2022 Oct 10;13(6):e02472-22. doi: 10.1128/mbio.02472-22 (PMC9765074; doi:10.1128/mbio.02472-22)
Supplement: FIG S6 [file mbio.02472-22-s0006.docx]

LkcD_ADN64230 -------FPGQGSQRIGMGKEVFD---AYPQLCDRADEI----VGHSLRELCLKDPDG--

BryP-AT1_ABM63531 -METIYLFPGQGSQHKEMGKYLFD---KYPELIHQADQQ----LHYSIKELCLEDPDQ--

OocV-AT1_AFX60344 -MKTVYTFPGQGSQYRTMGYGLFE---QFPELAAQASQA----LGYDIAELCIKDPQR--

**SolE-AT1_ANE74136** -----WVFPGQGSQQKGMGNELFE---RFPRLVSEADEI----LGFSIRELCLSDPQG--

RizA_CCA89325 ------IFPGQGSQHVGMGTGLFD---GAPDELAAADAV----LGWSVRELCLEDRDR--

RizF_CCA89330 -MTKIFVFPGQGAQKVGMGAELFR---EFPQEVAFADAI----LGYSIEELCLKDSEK--

PedD_CAE01104 --MKSYLFPGQGSQHLGMGEQLFD---RFPNIIEAANDI----LGYSIKTLCLEDPQR--

RhiG-AT2_YP_004029399 GPKRVYVFPGQGSQRVGMGAELFE---QFPDHVAQADEI----LGYSLRTLCLEDPDR--

LnmG_AF484556_42 --MVALVFPGQGSQRKGMGADLFA---RFPDLTRQADTV----LGHSVEELCRSSGDG--

SorO-AT2_ADN68489 ---IAFMFPGQGSQKRGMGAGLFDSVPQYRAVEKDVDAL----LGYSLRALCLEDPKN--

MmpC-AT2_AAM12912 QSKTVYMFPGQGSQFRGMGEGLFE---RFAELTACADRV----LGYSIRELCENDPRN--

OocW_AFX60345 -------FPGQGSQQPGMGRELFS---QFPDLTRIADDI----LGYSIERLCLDDPDG--

TaV-AT2_YP_632122 ----AALFPGQGSQERGMGAALFD---EFPDLTDIADAI----LGYSIKRLCLEDPGK--

KirCI-AT2_CAN89639 -----YVFPGQGAQVKGMGRDLFD---RFPELVERADAV----LGYSIRELCLEDPGR--

DisD_CAI43936 --MKAYMFPGQGSQAKGMGRALFD---AFPALTARADGV----LGYSIRALCQDDPDQ--

ChiA_YP_001614781 --MAVFVFPGQGAQRKGMGADLFD---RFRRVIGAADEI----LGYSIKELCLENPDQ--

PksC_NP_389591 --MITYVFPGQGSQKQGMGSGLFD---EFKELTDQADEI----LGYSIKRLCLENPYS--

BaeC_YP_001421285 --MITYLFPGQGSQKQGMGSSLFD---EFKDLTEQADET----LGYSMKRLCLENPYS--

PksE_CAB13584 --MITYVFPGQGSQQKGMGQGLFE---QYQHLTDQADQI----LGYSIEKLCTEKSYL--

BaeE_CAG23952 --MISFVFPGQGSQRIGMGEDLFG---RYPELTAKADHI----LGYSIQELCRDGE----

KirCII_CAN89643 ------VFAGQGAQWDGMGLELLDTEPVFGAALRRCDERVRELAGFSVIQQLRAGPAMS-

BryP-AT2_ABM63531 ------MYSGQGSQYYQMGKELYDNNSLFRHHMNYCSNQLKDRLGVSLIDIIYDKSKKNE

OocV-AT2_AFX60344 ------MFAGQGSQYYQMGRAFYHSDGHFRQTMDRLCHKVYMLSGVNLLEQLYSERDAAA

**SolE-AT2_ANE74136** ------MFGGQGAQYYGMGRELYRRNSTFRSQMDRCDALYRQHTGHGLLEALYDDARHHL

PedC_AAS47559 ------MFSGQGSQYFQMGRQLYEQDETFHAWMKSLDDNVRDYIGQSLLDIIYDTGHERS

SorO-AT1_ADN68489 ------MFSGQGSHYFQMGQELYERQATFRRWMDRLDVVTRDLSGASVVEALYKRGHGKG

RhiG-AT1_YP_004029399 ------MFPGQGCQFYQMGRELYQNNSVFHRWMNELDALIRVELGHSLIAEIYDANNARS

BaeD_YP_001421286 ------MFSGQGSQYYQMGKELFAHNAAFRQKMLDLDDFAVSRFGYSVLKEMYHTGNRLS

PksD_NP_389592 ------MFSGQGSQYYHMGKELFKENTVFRQSMLEMDAIAARRIGTSIVEEIYHPGKRVS

TaV-AT1_YP_632122 ------LFSGQGTQSYFMAKELFDTQTGFKRQLLELDEQFKQRLGHSILERIYDARAARL

MmpC-AT1_AAM12912 ------MFSGQGSQYRHMGRALYERHAGFGRHMRALDEVVRQTGGYSVLDGLYGDQPQAA

KirCI-AT1_CAN89639 ------LFAGQGSQYHGMGRWLYGADPFFRDALDSLDAVVREINGDSVIDAIHGDGRGAE

: *** : *. . .:

LkcD_ADN64230 -RLNETSRTQEAVYFVSCLMYLAYAEEHGAEQVRCLTGHSLGLYPALFAAGVFDLFEGLE

BryP-AT1_ABM63531 -LLNKTQFTQPALYIINALSFLEKIEL-DSHKPSYVAGHSLGEYNALFAAGAFDFLTGLK

OocV-AT1_AFX60344 -VLNQTQYTQPALYTVNALSYLERKAR-GAAPPDILAGHSLGEYNALFAAGAFDFITGLK

**SolE-AT1_ANE74136** -VLGETMFTQPALFVVSALGVLASRQS-GLDAPDCYAGHSLGEFAALFAAGAFDFATGVA

RizA_CCA89325 -KLNQTAWTQPALYVVNALAYLKKVRE-TGQRPALVAGHSLGEYNALFAAGAFDFITGLR

RizF_CCA89330 -RLHRTEFTQPALFVVNALAYLHAVER-NGTRPDFAAGHSLGEYNALFAAGAFDFITGLR

PedD_CAE01104 -QLRLTQYTQVALYVVNALTYRQHLQQ-GGGLPDFVAGHSLGEYNALESAGVFSFEDGLR

RhiG-AT2_YP_004029399 -QLSHTQYTQPALYTVNALAFLNKQQQ-DRRQPDYLAGHSLGEYCALFAAGAFSFETGLK

LnmG_AF484556_42 -RLDRTEYAQPALFVVSALSYLAR-DP-GLPQPTLLAGHSLGEYGALFAAGCFDFATGVR

SorO-AT2_ADN68489 -VLSDSQFTQPALYMVNALHYYQLLAE-GA-RPDYIAGHSLGEYNALHAAGAFDLLTGLR

MmpC-AT2_AAM12912 -ELGQTRFTQPALYVVNVLSYLAQAGD-AA-PPDYVLGHSLGEFCALFAAGAYDFETGLR

OocW_AFX60345 -ELNKTQYTQPAIYVVNALSYYKKRQE-TGIQPDFVLGHSLGEFNALLAAECFDFATGLK

TaV-AT2_YP_632122 -ELAQTQFTQPALYVVNALSYLKRLRE-GAEQPAFVAGHSLGEYNALLVAGAFDFETGLR

KirCI-AT2_CAN89639 -NLRDTRYTQPALYVVGALSWLATVQE-GGRLPDYLLGHSLGEFAALFAAGVYDFETGLR

DisD_CAI43936 -RLSQTQFTQPALYVVNALSYLKRREE-EA-PPDFLAGHSLGEFSALFAAGVFDFETGLA

ChiA_YP_001614781 -RLNQTQYTQPALFTINALSYYQRLED-QGVRPAYLAGHSLGEYSALLAAGAFDFETGLL

PksC_NP_389591 -NLNKTQFTQPALYVVNALSYLKKIRD-EEVKPDFVAGHSLGEYNALFAAEAFDFETGLQ

BaeC_YP_001421285 -NLHKTQFTQPALYVVNVLSYLKKIQD-NDIKPDYVAGHSLGEYNALFAAGAFDFITGLQ

PksE_CAB13584 -DVNHTEYTQPALYVVNALSYLKRVEE-TGRKPDFAAGHSLGEYNALMAAGAFDFETGLR

BaeE_CAG23952 -RLNQTQFTQPALYVVNALSYLKKTEE-TGLKPDFTAGHSLGEYNALYASGAFDFEEGLQ

KirCII_CAN89643 -RLGEIDVLQPTMVSLQIALVALWRSW-RV-EPDAVTGHSMGEISAGYAAGALTLDDALL

BryP-AT2_ABM63531 -EFDNIIYTNPVLYIFGYSLTQVLIDK-GI-KPDAFLGHSLGEYIAATVAGIISLEDGLN

OocV-AT2_AFX60344 -EFDDIRHTHPALFCFGYSLAQMLIAR-GI-QPAAVVGHSLGEYIAAVVAGMLRLDDALR

**SolE-AT2_ANE74136** -PFSDVALSNVALLSVGVSLTGMLKAE-GI-EPDAVLGYSLGECIAAVVAGVLTLDDAIK

PedC_AAS47559 LPFDRLIHTHPALFMVQYALAKSLLAR-GLPAPDFLIGASLGEFIAISLAGDTHVENILF

SorO-AT1_ADN68489 DLFDRTLLSHPALFMVEYALARALLEA-GV-EPGMVLGASLGTFTAATIAGCLDVEDGLK

RhiG-AT1_YP_004029399 KTFDDLRISHPAIFMVEYALGKTLIEQ-QI-QPDYLLGTSLGELAAAALAETLPLSDAIR

BaeD_YP_001421286 DPFDRLLFSHPAIFMAEYALAYALEQR-GI-RPDYVIGASLGEYAAAAVSGVLSAEDALD

PksD_NP_389592 DPFDSILFSHPAIFMIEYSLYKVLEDR-GI-YPDYVLGSSLGEFAAAAVSGVSDAEDMLD

TaV-AT1_YP_632122 DPLDDVLVSFPAIFMIEHALARLLIDR-GI-QPDAVVGASMGEVAAAAIAGAISVDAAVA

MmpC-AT1_AAM12912 -ALDDILVTHPAIFMVEYALAQSLIEH-GV-YPDHVLGSSLGEVAAAAVSGALDAEQALG

KirCI-AT1_CAN89639 LAMTRLSLTQPAIFMVEYALARMLRAH-GF-EPELVLGASLGEVVAAAVAGIFDPEECLR

. .: * *:* * : :

LkcD_ADN64230 IVSRRGALMQE----ARDGAMVAVLGPRASEIDDHLARLEFFDVDVANYNSPEQVVLSAL

BryP-AT1_ABM63531 LVKKRGLFMEE----APKGAMAAIIGITHNQVKCILEDIPQKNIDIANINSEKQFIISGL

OocV-AT1_AFX60344 LVQKRGQLMSL----APKGAMAAVLEINVADIERILAESGFQHVDIANINSLQQCIISGA

**SolE-AT1_ANE74136** LVRERGMLMSK----APRGAMAAIVGIDLHQIVELLAASPLSGIDIANINSAQQIVISGL

RizA_CCA89325 LVQRRAAVMAK----AREGGMAAVIGLSAARVREVLREAKLDEIDLANLNAPEQQVLSGP

RizF_CCA89330 LVQRRGQLMGR----ADGGTMAAVVGLSPEQVEQCLRVGGNDEVDIASLNAPEQTVISGA

PedD_CAE01104 LVQKRGDLMSQ----APRGAMAAILGISADSVAGILAEQGLTRIDIANYNAPTQTIISGL

RhiG-AT2_YP_004029399 LVKKRGELMAR----ATGGSMAAVIGRSADEIKSLLSQHGLNALDVANYNSPSQTVLAGP

LnmG_AF484556_42 LVRERGALMGR----AQGGGMLAVLGVDGDEVQALLAGTGARQVDVANYNTPTQTVLSGP

SorO-AT2_ADN68489 LVKRRGELMSQ----AKKGGMAAVIDMDERGIRKVLEENDLGTIDIANFNSPGQIVISGP

MmpC-AT2_AAM12912 LVKRRGELMSE----ATGGGMSAVLNLDLATIKQVLRQAGSTQLDFANFNAPQQTVLAGP

OocW_AFX60345 LVKKRGELMAQ----AAGGGMAAVLGLSEAKLRAFLTEKKLDTIDLANFNTPSQIVISGQ

TaV-AT2_YP_632122 LVKRRGELMSG----ASGGTMAAVVGCDAVAVEQVLRDRQLTSLDIANINSPDQIVVSGP

KirCI-AT2_CAN89639 LVAERGRLMGQ----VTGGTMAAVSAVDSSLVREVLRDDELSGLDIANYNAPTQTVVAGP

DisD_CAI43936 LVKKRGELMGD----ARGGGMAAVIGLDEERVRELLDQNGATAVDIANLNSPSQVVISGA

ChiA_YP_001614781 LVKKRGELMSK----ADKGGMAAVFGLREDDILEALQRHGLRRLHIANHNTPSQIVISGA

PksC_NP_389591 LVRKRGELMSL----ISNGGMAAVMGLNEEQVAKALKEYHLHDVDIANVNAPYQIVISGK

BaeC_YP_001421285 LVRKRGELMSM----ATDGKMAAVMGLTAAQVSDALQTHGLHTIDIANMNSPHQVVISGR

PksE_CAB13584 LVKKRGELMGR----ITGGGMAAVIGLSKEQVTAVLEEHRLYDIDVANENTPQQIVISGP

BaeE_CAG23952 LVKKRGELMSR----AKGGGMAAVIGLTHEQVTDVLRENHLDMIDIANMNTPQQIVISGY

KirCII_CAN89643 IACRRSALLRRI---AGRGALATTELSPEAAHA--LAASSGGRICVAGENSPRSTVLAGD

BryP-AT2_ABM63531 LILTQAQLLEKH---CNTGKILNVFSPPDVYYK---NQNLFFNTPLACVNFSNNFSVSGY

OocV-AT2_AFX60344 LVVEQAHLLEQH---APAGRLAVVLAAPDIFHR---ATEVFSRCTLAGVNFGNNFFISGE

**SolE-AT2_ANE74136** LVVSQAHLLHKK---TAGGGMMSVLAPVAHFHA---NAALYQGAELASINFQNNFVVSGD

PedC_AAS47559 NLIKQARLFDEY---CNAGAMLLVIDHIDTFST---TPAFSKDCELAGINFDHCFVVSGP

SorO-AT1_ADN68489 AAIQHAKALEST---CEPGAMIAVLAPLRLCEDASLHRYG----ELAAVNFDGNFVLSVR

RhiG-AT1_YP_004029399 FVTRQGQLFHRRQSPATEGTMLAILCDESLYQQ---TPLLHDHCDIAAYNAQSLIVIAGQ

BaeD_YP_001421286 CVLEQARIVTET---CRNGSMLAILGDPALYQD---DPLLGEHSELASVNYHSHFVISGE

PksD_NP_389592 CILEQAIIIQNS---CDKGKMLAILDKPQLLND---HPQLFGNSELISINYDSHFVISGE

TaV-AT1_YP_632122 LVAAQAQLFART---APRGGMLAVLHELEACRG---FTSVARDGEVAAINYPSNFVLAAD

MmpC-AT1_AAM12912 FVVRQARLFHEH---CPAGGMTAVLADIAVFEQ---LQADGIEVELAAINYPQHLVISGT

KirCI-AT1_CAN89639 SLLEQVALFEAE---CPRGGMLAVLADAGLVDR----EPALAGAHLAAINGPDNFVLAGT

. . * : . * ::

LkcD_ADN64230 KPRLEELVPRLEE-TGHR-CVWLPVSGAFHSRHMEPARLRFAQFLRDRTFTPPTKPVVST

BryP-AT1_ABM63531 YDEIIACENSFTK-MGAN-FILLNVSAAFHSRYMKDIEIKFEQYLQKFQLNPLRTPVISN

OocV-AT1_AFX60344 YDEVLALEPRFLE-AGAR-FVQLNVSAAFHSRLMSDIEREFADYLTQFTFQPLSTPVLSN

**SolE-AT1_ANE74136** YDDIVACETLFTD-AGAR-YVRLNVSAAFHSRYMRDIEAQFAQFAQRFTFNPLNARVISN

RizA_CCA89325 VESLVQAEAVFTR-VGVRGFKRLPVSAAFHSRYMREARQEFESFLEEFVLSSPRIPVISN

RizF_CCA89330 ASAFERVEAALTA-AGAREVVRLRVSAAFHSRYMKKAEAEFASFLSQFEFSSLDFPVVSN

PedD_CAE01104 EADIRDAQAVFES-CQAM-YVPLNTSGAFHSRYMQSARDEFAQFLEAFEFRDPQIPVVAN

RhiG-AT2_YP_004029399 VEALSQAKEIFQA-LEIT-VIPLSVSAPFHSRYMQSAMEEFGEYLKQFSYSPLQIPVISN

LnmG_AF484556_42 LDELRMVSAALGQRPGVR-CVPVRVSAAFHSRHMRPAAQEFATFLTGFSFADPHRTVISS

SorO-AT2_ADN68489 LDDISRAKSIFER-SGARSFIPLPVGAAFHSRYVADAARAFADFLAPMTFAPLQLPVISN

MmpC-AT2_AAM12912 LDALESVRTQIEDASGI--CVALNVSAPFHSRYMRGAQEAFAAELARVTFKPLTLPVIAN

OocW_AFX60345 RDDVTKAVTEFEQ-SGMR-CVPLNTSGAFHSRLMRGSMDKFESYLQNFQFSALKIPVIAN

TaV-AT2_YP_632122 AQDIERARQCFVD-RGAR-YVPLNVRAPFHSRYMQPAASEFERFLSQFQYAPLRCVVISN

KirCI-AT2_CAN89639 ADAVNRALAVFKD-KGAR-CAPLNVSAPFHSRYMAQAAEEFGRLLDATAFAAPKIPVISN

DisD_CAI43936 KDEIARLQVPFEA-AGAKKYTVLRVSAAFHSRFMRPAMVEFGRFLEGYDFAPPKIPVISN

ChiA_YP_001614781 AEEIQRAKPVFDGIAGAR-YVPLNVSGAFHSPLMAEAREEFAAYIARFDLRNPGIPVVSN

PksC_NP_389591 KDEIEKAASLFETMTEVTMVLPLNVSGAFHSRYMNKAKEEFEEFLHAFYFSPPSIPVISN

BaeC_YP_001421285 KEDIERAKSVFEGLKDVTMFHPLNVSGAFHSRYMSEAKQEFEKFLQSFHFSAISIPVISN

PksE_CAB13584 KKEIEKARAVFENTKDVKLFHPLNVSGAFHSRYMNEAKQVFKQYIDSFQFAPLAIPVISN

BaeE_CAG23952 KEDIEKAASVFEAVNGVKMVHRLNVSGAFHSRYMLEAKEEFSRFIESFHFKPLSIPVISN

KirCII_CAN89643 TATLTALVEDLDR-RGVY-CRMVRGTVASHSHYVDELRDDLAGALRPLSPVPSRVPFYST

BryP-AT2_ABM63531 RYEIDLVKKELDK-RKIF-SSYLPVSHGFHSHAIDPIENYFKKYVSQIKYSANKLPIYSC

OocV-AT2_AFX60344 QSAVTSAIQKLTE-RDIL-SLSLPVRYAFHSASIDAIKPSYQKLLDKTPCFAPTMPVYSS

**SolE-AT2_ANE74136** VASLSALKERLTQ-LEII-SMLLPVEQPFHSSGIAAIEHDFRALVDTLPKNTPAMPVYSA

PedC_AAS47559 RTGILQTRKSLTK-QNIA-CQLLPVSIAFHSSWMDEVHEIFIQQFPEQICRRLHTPVISC

SorO-AT1_ADN68489 THDVSAVQEILRG-RGVT-FRRLPVSFAFHSRWMDPAETSFKRHMRSVITRRGDLPLVCC

RhiG-AT1_YP_004029399 SARIAEAERYLSS-RDIV-FQRLPVKQAFHSRHIDFLKPEVDVLASELRLRRAQIPVISC

BaeD_YP_001421286 REHIKKIMDDLRE-KQIP-HQLLPVSYGFHSALVDQAEQPYKRFLAQKSIRTPFIPYISS

PksD_NP_389592 EDHIRKIMEDLKE-KQIL-CQLLPVSYAFHSSLIDPAESAYAEFLRSKSFQKPSIPIVSS

TaV-AT1_YP_632122 EAGLGRIQQELSQ-RSVA-FHRLPVRYPFHSSHLDPLREEYRSRVRADSLTWPRIPMYSC

MmpC-AT1_AAM12912 VSALAAAQMQLAR-RNVV-FQRLDVNRPFHSSHMDPLRDAFIQTSQALQLRTPRMGYVSS

KirCI-AT1_CAN89639 AGRLDAIERHLAA-SGVL-CQRLPVLFPFHSPLIDGVRDAFTKLVGGLTPRAASIPLISG

. : : ** : .

LkcD_ADN64230 TSGRTLGARH----LLE-EMVFQLVKPVRWWQTVTHLSRT-----GHKTFDEVGPGRVLT

BryP-AT1_ABM63531 YSARPYPKEN----YRD-YMVKQISHPVKWYESISWLIQQ-----DHFEFEEVGPGRVLT

OocV-AT1_AFX60344 VTARAYPRTD----YQT-LLTRQISSPVRWYESISWLLAQ-----GCDDFAEVGPGDVLT

**SolE-AT1_ANE74136** YTALDYPETD----YLD-LLTRQISHPVRWYESISRLLAS-----GEVKQHEIGPGQVLT

RizA_CCA89325 VEAKPYPPGA----LKR-LLVEQITSPVRWEESVQYLLQQ-----PGPDLEEVGPGRVLT

RizF_CCA89330 LHAALYAPDQ----IHA-CLAGQISKPVRWTETIQLLARQ-----PEPVFEELGPSKFLT

PedD_CAE01104 VTAKPYVGTE----VVR-TLADQLTGSVRWLDSMRFLLDQ-----GVTEFRELGPGDVLS

RhiG-AT2_YP_004029399 IHAVPYRDNE----LID-NLTRQICGSVRWVETVQYLIRQ-----GECEFEELGPGNVLT

LnmG_AF484556_42 VTARPYGAGQ----VAE-LLSRQIESPVRWSETMAYLRER-----GTTELEEMGPGKVLT

SorO-AT2_ADN68489 VTARPYEAGNPSVAIKS-LLVEQITRPVRWMQSVEYLIDK-----GVRDFREVGPGNVLT

MmpC-AT2_AAM12912 VDARPYEQEA----IAS-QLARQMTSSVQWVESIEYLLQA-----GITQFKEIGPGNVLT

OocW_AFX60345 TTARPYEDDT----LLT-CLAQQIASPVRWTASIQYLMSL-----GTPEFTELGHSEVVS

TaV-AT2_YP_632122 VTGRPYAHDN----VVQ-GLALQLRSPVQWTATVRYLLEQ-----GVEDFEELGPGRVLT

KirCI-AT2_CAN89639 VDARPYEPDA----VAA-TLRRQIVSPVRWTDSIRLLMGR-----GDFRVRELGPGQVLT

DisD_CAI43936 VTARPCKADG----IRA-ALSEQIASPVRWCESIRYLMGR-----GVEEFVECGHGIVLT

ChiA_YP_001614781 VTARPYVDGR----ASQ-LLTEQITSSVQWTDSIRYLMGL-----GETDFKEIGPGNVLT

PksC_NP_389591 VYAKPYTYEF----MKQ-TLADQINHSVKWTDSISYLMKK-----GHMEFEEVGPGNVLT

BaeC_YP_001421285 VHARPYEQDG----IHS-VLADQIDHSVRWNDSIRYLLDK-----GRMEFEEVGPGHVLT

PksE_CAB13584 VYAEPYHQDR----LKD-TLSEQMDNTVKWTDSIRFLMGR-----GEMEFAEIGPGTVLT

BaeE_CAG23952 VTARPYEQRE----LKE-TLTGQITGSVNWTDSIRFLMGR-----KNMSFEEIGPGKVLT

KirCII_CAN89643 VTAAPVPGTD----LGPAYWMRNLREPVRLAAATGRLAED-----GHEIFVEVSTHPVLL

BryP-AT2_ABM63531 YYTSELTQDNIDN-MKN-YLWEVIRNTINFEKLISSSFQNT----SNNIFIDTSPNAALS

OocV-AT2_AFX60344 VLGRAVGEMQ--QADAQGYLWQVIRDKVDFSTLVDSVFSTM----SDHFFIDASATGSLS

**SolE-AT2_ANE74136** MLSTAVERWD-----SE-YFWRVLREPVDFYGLMQALAEK-----NEAFFVDLSPTGTLS

PedC_AAS47559 ALPVPEQLTR----FSSTYWWHVIRQPIAFHLAINTFHQSS----PNAVYLDLGPAGNMA

SorO-AT1_ADN68489 ARSQPLDALS-----DE-RLWRATRDPIRFQDTLRRLEAS-----GPHQYVDMDPAGTLA

RhiG-AT1_YP_004029399 HNTETLNQLT-----TD-HFWRTIREPIRFSQTLARIEREAAERGESMIYLDLGPSGTLA

BaeD_YP_001421286 ATGEAETDIQ-----AD-FFWDIVRKPIRFREALQFADSR-----QKGLYIDAGPSGTLA

PksD_NP_389592 LTGSCLHVMD-----EN-FFWNAVRKPMMFREAIRYLESQ-----HTCKFIDLGPSGTLA

TaV-AT1_YP_632122 TTANRVHDLR-----SD-HFWNVVRAPIQLYDTVLQLEGQ-----GGCDFIDVGPAASFA

MmpC-AT1_AAM12912 TLGGPVSQFA-----PE-HLWHVVRSTLQLAPAIEYLEGL-----GPHEYLDAGPSGSMA

KirCI-AT1_CAN89639 TTGAEVRHPG-----PD-HFWQVLREPFDLSRALEPLLAR-----DDLLFLDLGPSGSMA

. : . .

LkcD_ADN64230 KLSAEILGD-------------

BryP-AT1_ABM63531 NLTNQIKKTP------------

OocV-AT1_AFX60344 KLHQRI----------------

**SolE-AT1_ANE74136** SLFDKIK---------------

RizA_CCA89325 TLVAQIRRK-------------

RizF_CCA89330 PLIKSTVK--------------

PedD_CAE01104 KLVESIRSSAMSKPVSEFAA--

RhiG-AT2_YP_004029399 KLINSI----------------

LnmG_AF484556_42 GLWKQ-----------------

SorO-AT2_ADN68489 RLVNKIHKLA------------

MmpC-AT2_AAM12912 NLQAKIEKN-------------

OocW_AFX60345 GLVEKIKAETT-----------

TaV-AT2_YP_632122 RLITANKRG-------------

KirCI-AT2_CAN89639 KLIARIRDEA------------

DisD_CAI43936 GLYAQIRRDAQPLVVDEGAA--

ChiA_YP_001614781 KLVQQIKQEAT-----------

PksC_NP_389591 GLIHRIKKDAEA----------

BaeC_YP_001421285 GLIHRIKNETEASP--------

PksE_CAB13584 GLIHRIKNEAE-----------

BaeE_CAG23952 GLIQRITAEAEPITDE------

KirCII_CAN89643 SSLRQTLESAGRPGEVLPSGRR

BryP-AT2_ABM63531 NSLK------------------

OocV-AT2_AFX60344 NFLK------------------

**SolE-AT2_ANE74136** IFIK------------------

PedC_AAS47559 AATKYNLPSSIHYRILPTM---

SorO-AT1_ADN68489 TFVKYNLAQTPSASKFHAVFS-

RhiG-AT1_YP_004029399 NLIKQNIRDRQAPQTFAVLSP-

BaeD_YP_001421286 AFAKQILPAGSA----------

PksD_NP_389592 AFVKQLIPGDSAD---------

TaV-AT1_YP_632122 TIIKRILARDSTSRLFPLLSP-

MmpC-AT1_AAM12912 NFAKRCRSAHSAST--------

KirCI-AT1_CAN89639 NLVRARLPEGSRSRVLPLLSP-

**Supplementary Figure S6: Multiple sequence alignment of *trans-*acyltransferase (AT) domains.** S-H catalytic dyads are shown in red. AT active sites (blue) together with additional conserved residues (yellow) for malonate-specific domains and N-terminal GQGSP loop (underlined) based on Yadav and co-workers (2003) and Keatinge-Clay and coworkers (2003) are shown. Leucine and isoleucine residues typically found in AT domains selective for methylmalonyl-CoA are shown in pink (Keatinge-Clay *et al*., 2003). Protein GenBank accession numbers are also shown.
